# Molecular Diversity of Clinically Stable Human Kidney Allografts

**DOI:** 10.1001/jamanetworkopen.2020.35048

**Published:** 2021-01-25

**Authors:** Dmitry Rychkov, Swastika Sur, Marina Sirota, Minnie M. Sarwal

**Affiliations:** 1 Division of Multi-Organ Transplantation, Department of Surgery, University of California, San Francisco; 2 Bakar Computational Health Sciences Institute, University of California, San Francisco; 3 Department of Pediatrics, University of California, San Francisco

## Abstract

**Importance:**

Clinical decision and immunosuppression dosing in kidney transplantation rely on transplant biopsy tissue histology even though histology has low specificity, sensitivity, and reproducibility for rejection diagnosis. The inclusion of stable allografts in mechanistic and clinical studies is vital to provide a normal, noninjured comparative group for all interrogative studies on understanding allograft injury.

**Objective:**

To refine the definition of a stable allograft as one that is clinically, histologically, and molecularly quiescent using publicly available transcriptomics data.

**Design, Setting, and Participants:**

In this prognostic study, the National Center for Biotechnology Information Gene Expression Omnibus was used to search for microarray gene expression data from kidney transplant tissues, resulting in 38 studies from January 1, 2017, to December 31, 2018. The diagnostic annotations included 510 acute rejection (AR) samples, 1154 histologically stable (hSTA) samples, and 609 normal samples. Raw fluorescence intensity data were downloaded and preprocessed followed by data set merging and batch correction.

**Main Outcomes and Measures:**

The primary measure was area under the receiver operating characteristics curve from a set of feature selected genes and cell types for distinguishing AR from normal kidney tissue.

**Results:**

Within the 28 data sets, the feature selection procedure identified a set of 6 genes (*KLF4*, *CENPJ*, *KLF2*, *PPP1R15A*, *FOSB*, *TNFAIP3*) (area under the curve [AUC], 0.98) and 5 immune cell types (CD4^+^ T-cell central memory [Tcm], CD4^+^ T-cell effector memory [Tem], CD8^+^ Tem, natural killer [NK] cells, and Type 1 T helper [T_H_1] cells) (AUC, 0.92) that were combined into 1 composite Instability Score (InstaScore) (AUC, 0.99). The InstaScore was applied to the hSTA samples: 626 of 1154 (54%) were found to be immune quiescent and redefined as histologically and molecularly stable (hSTA/mSTA); 528 of 1154 (46%) were found to have molecular evidence of rejection (hSTA/mAR) and should not have been classified as stable allografts. The validation on an independent cohort of 6 months of protocol biopsy samples in December 2019 showed that hSTA/mAR samples had a significant change in graft function (*r* = 0.52, *P* < .001) and graft loss at 5-year follow-up (*r* = 0.17). A drop by 10 mL/min/1.73m^2^ in estimated glomerular filtration rate was estimated as a threshold in allograft transitioning from hSTA/mSTA to hSTA/mAR.

**Conclusions and Relevance:**

The results of this prognostic study suggest that the InstaScore could provide an important adjunct for comprehensive and highly quantitative phenotyping of protocol kidney transplant biopsy samples and could be integrated into clinical care for accurate estimation of subsequent patient clinical outcomes.

## Key Points

**Question:**

Can immunologic heterogeneity be identified in histologically stable kidney allografts?

**Findings:**

This prognostic study used 28 public kidney transplant data sets with 2273 kidney tissue to develop and validate an unbiased, 6-gene and 5-cell-type transcriptional Instability Score to provide histology-independent reclassification of human transplant samples. Using this score, 46% of histologically stable samples were found to have molecular evidence of rejection, which was validated by an independent cohort that showed undiagnosed graft rejection and poor projected graft function survival.

**Meaning:**

These findings suggest that the Instability Score could provide an important adjunct for comprehensive and highly quantitative phenotyping of protocol kidney transplant biopsy samples and could be integrated into clinical care for accurate estimation of subsequent patient clinical outcomes.

## Teaser

This prognostic study uses microarray gene expression data from the National Center for Biotechnology Information Gene Expression Omnibus to assess whether immunologic heterogeneity can be identified in histologically stable kidney allografts.

## Introduction

Breakthroughs in surgical approaches and development of newer generations of immunosuppressive drugs have resulted in reduction of clinical allograft acute rejection (AR) and improvements in life expectancy and quality of life for kidney transplant recipients.^1^ Nevertheless, a burden of subclinical AR is present only at a molecular level, not associated with an alteration in graft function, and often not accompanied by changes in graft histology.^2,3,4,5,6,7,8^ In addition, the significant discrepancies (19%-55%) among pathologists for histologic phenotyping^9,10^ result in a lack of consistency in interpreting an allograft as rejected,^11,12^ not rejected, or stable,^7,9,10,13^ thereby introducing bias in the interrogative mechanistic studies on allograft pathology. Furthermore, there is a failure to uncover the molecular biologic diversity in the histologic definition of a stable allograft. This bias is further amplified during interrogation of kidney transplant biopsy samples across different pathologists and investigators in public data sets.

In this study, we have aggregated, to our knowledge, the largest public data set for human kidney transplantation to date: 2273 kidney tissue microarray samples from 28 publicly available normal and transplant kidney tissue data sets^14^ in Gene Expression Omnibus,^15^ a public genomics data repository, to investigate the molecular diversity of stable allografts.^16,17,18,19^ We proposed that for accurate definition of a stable allograft, the sample must be associated with (1) stable clinical function, (2) normal kidney histology with AR (histologically stable [hSTA]), and (3) absence of a transcriptional signature of AR (molecularly stable [mSTA]). Recognizing the previously discussed variabilities in allograft histology interpretation, we expected that some of the labeled stable samples in these data sets (that only use the first 2 criteria listed above) would have inherent molecular variability. Our analysis has resulted in the generation of a histology-independent composite gene and cell-specific computational Instability Score (the InstaScore) to discern molecular rejection in hSTA allografts, classifying clinically stable (truth) samples as histologically and molecularly stable (hSTA/mSTA) or clinically and histologically stable (untruth) samples with molecular rejection (hSTA/mAR). Thus, our prognostic study proposes an approach to recognize immunologic heterogeneity in hSTA kidney allografts.

## Methods

### Data Collection

For this prognostic study, we carried out a comprehensive search for publicly available microarray data at the National Center for Biotechnology Information Gene Expression Omnibus database^15^ for biopsy kidney transplant samples from January 1, 2017, to December 31, 2018. Any public, deidentified data available as open access were not subject to local institutional review board requirements or patient consent as allowed under the Common Rule. For any private data used, we obtained the approval of the institutional review board of the University of California, San Francisco, and written informed consent from all patients. After stringent data quality control procedures (eMethods in the Supplement), the final data set consisted of 28 studies with 2273 samples. Their diagnostic annotations included 510 AR samples (including antibody-mediated rejection, T-cell–mediated rejection, AR, AR with chronic allograft nephropathy, borderline rejection, borderline rejection and chronic allograft nephropathy, and mixed rejection), 1154 stable samples, and 609 normal samples (ie, biopsy conducted before organ transplant). The summary for the collected studies is represented in eTable 1 in the Supplement. This study adhered to the Preferred Reporting Items for Systematic Reviews and Meta-analyses (PRISMA), Standards for Reporting of Diagnostic Accuracy (STARD), and Transparent Reporting of a Multivariable Prediction Model for Individual Prognosis or Diagnosis (TRIPOD) reporting guidelines.

### Data Processing and Normalization

Raw fluorescence intensity data were downloaded and preprocessed depending on the microarray platform. The data processing included background correction, log2 transformation, quantile normalization, and probe to gene mapping using R language, version 3.5.1^20^ (R Foundation) (eMethods and eFigure 1A in the Supplement). To perform a meta-analysis, we merged all the studies and corrected for potential batch effects using the ComBat^21^ approach (eFigure 2 in the Supplement); however, other approaches were evaluated (eMethods in the Supplement).

### Statistical Analysis

To identify differentially expressed genes, we used the Significance Analysis of Microarrays,^22^ which used the siggenes package.^23^ We used the false discovery rate^24^ with the Benjamini-Hochberg^25^ method for multiple testing correction (*P* < .05 and FC > 1.5).

### Pathway Analysis

We leveraged the Gene Ontology database using the gene set enrichment analysis with clusterProfiler^26^ to perform functional annotations for the significantly upregulated and downregulated genes with a false discovery rate less than 0.05. For the gene network analysis, we used the STRING protein-protein association networks database.^27^

### Cell Type Enrichment Analysis

To estimate the presence of certain cell types in biopsy samples, we used the cell type enrichment tool xCell.^28^ xCell leverages gene expression data from microarray or RNA-sequence experiments to estimate the presence of up to 64 immune and stromal cell types in a mixture. We focused on 34 immune-related and 11 nonimmune cell types (eTable 3 in the Supplement) that were manually selected as relevant to the transplant injury process. The enrichment scores for each cell type were used to compare AR and normal samples by performing the nonparametric 2-sample Mann-Whitney-Wilcoxon statistical test. The *P* values were adjusted using the Benjamini-Hochberg method (*P* < .05).

### Feature Selection Procedure

In order to select the most important features in distinguishing AR vs normal samples, first, the data were split into training and testing sets in the ratio 80:20. All feature selection steps were performed on the training set with benchmarking on the testing set. Among the significant features, we searched for features correlated with the outcome (*r* > 0.75 × max[*r*]). After, we applied the recursive feature elimination technique with the random forest (RF) model using caret.^29^ We used a 5-fold cross-validation technique with 100 repeats and benchmarked a model by computing the area under the receiver operating characteristic (AUROC) curve, and the results were reported with both AUROC and precision-recall area under the curve (AUCPR). To minimize possible bias of the data random split and to avoid the model overfitting, the tolerance of 1% to the feature selection mechanism was introduced, ie, the algorithm chose a model with a smaller number of features that performed no worse than 99% from the best model. A final set of selected features was benchmarked by applying the RF model to the testing set. The R package feseR^30^ was adopted and modified for the implementation of the parallel computations.

### Instability Score and hSTA Subphenotyping

The method of subphenotyping hSTA samples was based on selected features from the normal or AR analysis and applied for scoring the hSTA samples. The hSTA samples were then identified as mAR or mSTA. We denoted this split as hSTA/mAR and hSTA/mSTA, respectively.

Based on gene expression and cell type enrichment data, the feature selection procedure was performed to find sets of genes and cell types highly associated with AR. Next, with Z-scaled features, we built a logistic regression model and, using model coefficients, created a linear score function, the InstaScore:

InstaScore = 0.596 + 2.096 × *KLF4* + 2.534 × *CENPJ* + 0.311 × *KLF2* + 1.447 × *PPP1R15A* – 1.633 × *FOSB* + 0.268 × *TNFAIP3* + 2.249 × natural killer (NK) cells +0.542 × CD4^+^ T-cell central memory (T_cm_) cells +0.833 × CD4^+^ T-cell effector memory (T_em_) cells +0.709 × CD8^+^T_em_ cells +0.146 × Type 1 T helper (T_H_1) cells

Therefore, the positive InstaScore values separate AR from normal samples, which obtain negative values (eFigure 1B in the Supplement). Using this definition, the InstaScores were computed for the hSTA samples, and the zeroth threshold was applied to perform the split into mAR and mSTA subtypes (eFigure 1C in the Supplement). All the code has been uploaded to github.^31^

## Results

From the total 28 data sets, the feature selection procedure identified a set of 6 genes (*KLF4*, *CENPJ*, *KLF2*, *PPP1R15A*, *FOSB*, and *TNFAIP3*) (AUC, 0.98) and 5 immune cell types (CD4^+^ Tcm, CD4^+^ Tem, CD8^+^ Tem, NK cells, and T_H_1 cells) (AUC, 0.92) that were combined into 1 composite InstaScore (AUC, 0.99). We leveraged all currently publicly available kidney biopsy microarray data (eFigure 1A in the Supplement) from 28 studies with 2273 samples and performed a feature selection procedure based on the RF algorithm to identify a subset of genes and cell types that better distinguish AR and normal kidney samples, which were combined into the InstaScore (eFigure 1B in the Supplement) to reclassify all annotated stable samples (hSTA) and identify variances in the samples by recognizing similarities to either the molecular rejection signature (hSTA/mAR) or the molecular quiescence (hSTA/mSTA) (eFigure 1C in the Supplement). The clinical validity and prediction performance of the InstaScore were demonstrated on independent data wherein falsely classified stable samples (hSTA/mAR) showed significant projected differences in reduced graft function and survival over the true stable samples (hSTA/mSTA).

### Differential Gene Expression Analysis for Upregulation of Immune-Related Pathways in Rejection

**Figure 1.**
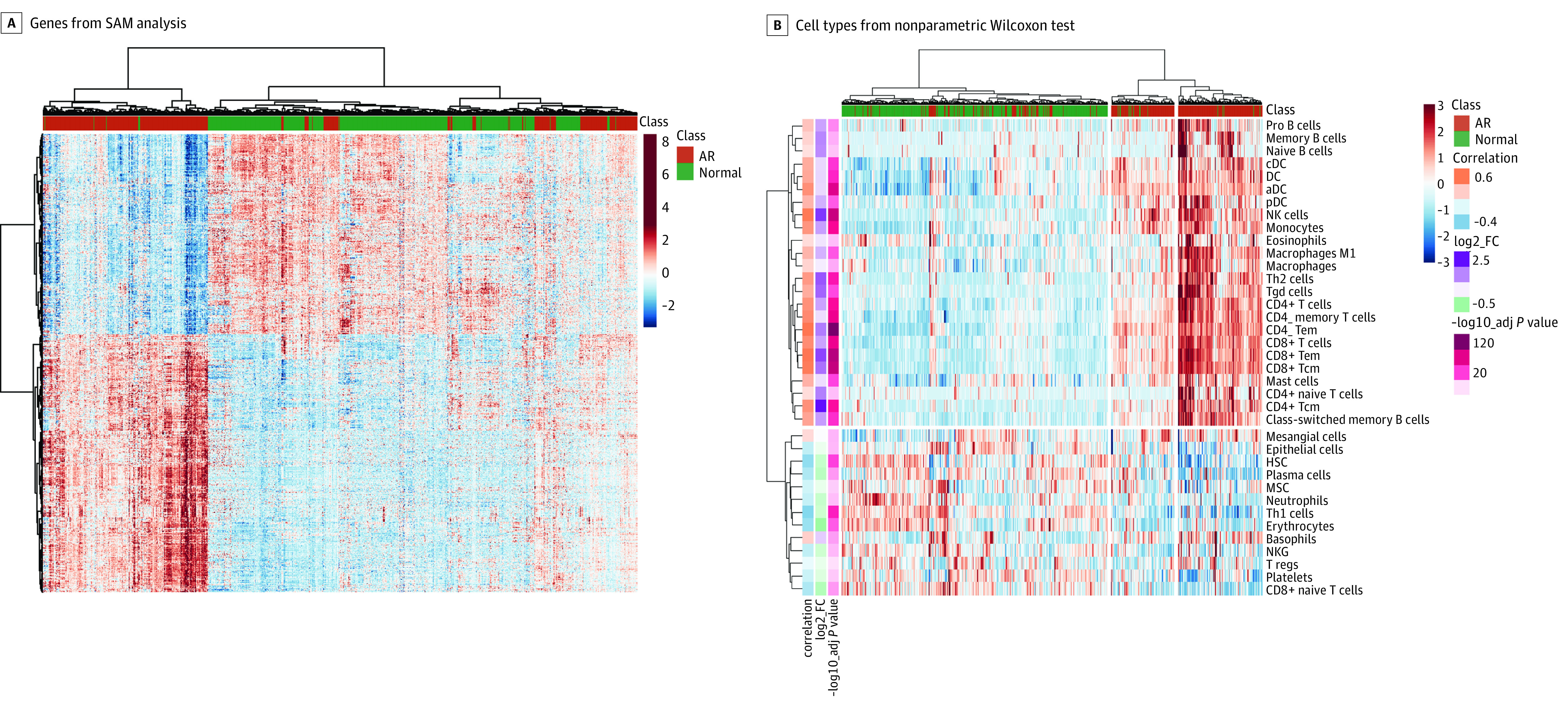
Heat Map Plots for Differentially Expressed Genes and Significantly Enriched Cell Types A, Heat map clustering plot for significant genes from Significance Analysis of Microarrays (SAM) of acute rejection (AR) vs normal samples. B, Heat map clustering plots for significant cell types from the nonparametric Wilcoxon statistical test (Benjamini-Hochberg, *P* < .05) in the analysis of AR vs normal samples. aDC indicates activated dendritic cell; cDC, conventional dendritic cell; DC, dendritic cell; FC, X; HSC, hematopoietic stem cell; M1, X; MSC, mesenchymal stem cell; NK, natural killer; NKG, X; pDC, plasmacytoid dendritic cell; Tcm, X; Tem, X; Tgd, X; Th1, Type 1 T helper cell; Th2, Type 2 T helper cell; T regs, regulatory T cell.

We performed differential gene expression analysis comparing AR with normal samples and identified 1509 significantly differentially expressed genes including 848 upregulated and 661 downregulated genes (eTable 2 in the Supplement). Further hierarchical clustering analysis on the significant genes based on the Ward clustering technique was performed, and a significant separation was found^32^ (1119 samples, 1509 genes; *P* < .001) between classes (Figure 1A). Additionally, the principal component analysis and Uniform Manifold Approximation and Projection dimensionality reduction confirmed the class separation (eFigure 3 in the Supplement). The functional annotation of the significant genes found that upregulated genes were enriched in the regulation of the immune response, cell aggregation and activation, and innate immunity (eFigure 4A in the Supplement). The downregulated genes were enriched in metabolic processes (eFigure 4C in the Supplement). The network analysis showed connectivity between the sets of genes (eFigure 4B and 4D in the Supplement).

### Cell Type Enrichment Analysis for Immune Cell Types Associated With AR

To highlight the biologic heterogeneity and to capture signals from infiltrating cell type–specific outcomes in injured and stable kidney transplants, we performed cell type enrichment analysis. We leveraged xCell^28^ to focus on 45 cell types (eTable 3 in the Supplement) that are relevant for organ transplants. We found 25 cell types (mostly lymphoid and myeloid cells) that were significantly (Wilcoxon test, Benjamini-Hochberg; 1119 samples; *P* < .05) enriched in AR and 12 cell types (immune, stromal cells, and hematopoietic stem cells) that were enriched in normal kidneys (Figure 1B). As seen on the heat map, the hierarchical clustering revealed 2 main AR subclusters (510 samples; *P* < .001): one was mostly enriched in lymphocytes, NK cells, and macrophages, and the other had minimal lymphocyte activation and may have represented temporal differences in rejection evolution or recovery. We observed that B cells, dendritic cells, macrophages, and T cells formed cell type–specific subclusters that suggested the coordinated activation of immune cells in the kidney tissues. These results are in agreement with previous observations^33^ that have shown AR subphenotypical splits by gene expression and cell type. Unsupervised clustering of hSTA along with AR and normal samples exposed their heterogeneity, hinting that some hSTA samples have molecular signal closer to AR samples (eFigure 5 in the Supplement).

### Machine Learning Feature Selection Procedure to Optimize AR Classification

Following the feature selection procedure (eMethods in the Supplement), we dramatically decreased the number of model features from all 1509 differentially expressed genes (1) to only 6 pivotal upregulated genes (*KLF4, CENPJ, KLF2, PPP1R15A, FOSB*, and *TNFAIP3*; AUROC, 0.98; AUCPR, 0.99) (eFigures 6A and 7A in the Supplement); (2) to genes enriched as zinc finger proteins and expressed mostly in CD33^+^ myeloid cells; and (3) to 5 cell types from the original set of 37 significantly enriched cell types: CD4^+^ Tcm, CD4^+^ Tem, CD8^+^ Tem, NK cells, and T_H_1 cells, with CD4+ Tcm having the largest effect size in this model (AUROC, 0.92; AUCPR, 0.88) (eFigures 6B and 7B in the Supplement).

The feature selected cell types showed a predominant role for infiltration and activation of effector T cells and NK cells in AR, and the feature selected genes appeared to have broad cellular functions in AR, triggered by mononuclear activation and infiltration and collectively driving a variety of functions, such as DNA recognition, RNA packaging, transcriptional activation, and regulation of apoptosis. Interestingly, although the set of 11 genes in the common rejection module previously identified from a cross-organ (kidney, heart, liver, and lung) meta-analysis study of transplant rejection^16^ was enriched in this current analysis, none of them made it to this final minimal feature selection set. This finding suggests that the current 6-gene set might be more specific for the absence of AR in the kidney allograft, as the precise definition of a hSTA/mSTA allograft was not available in the earlier analysis.

**Figure 2.**
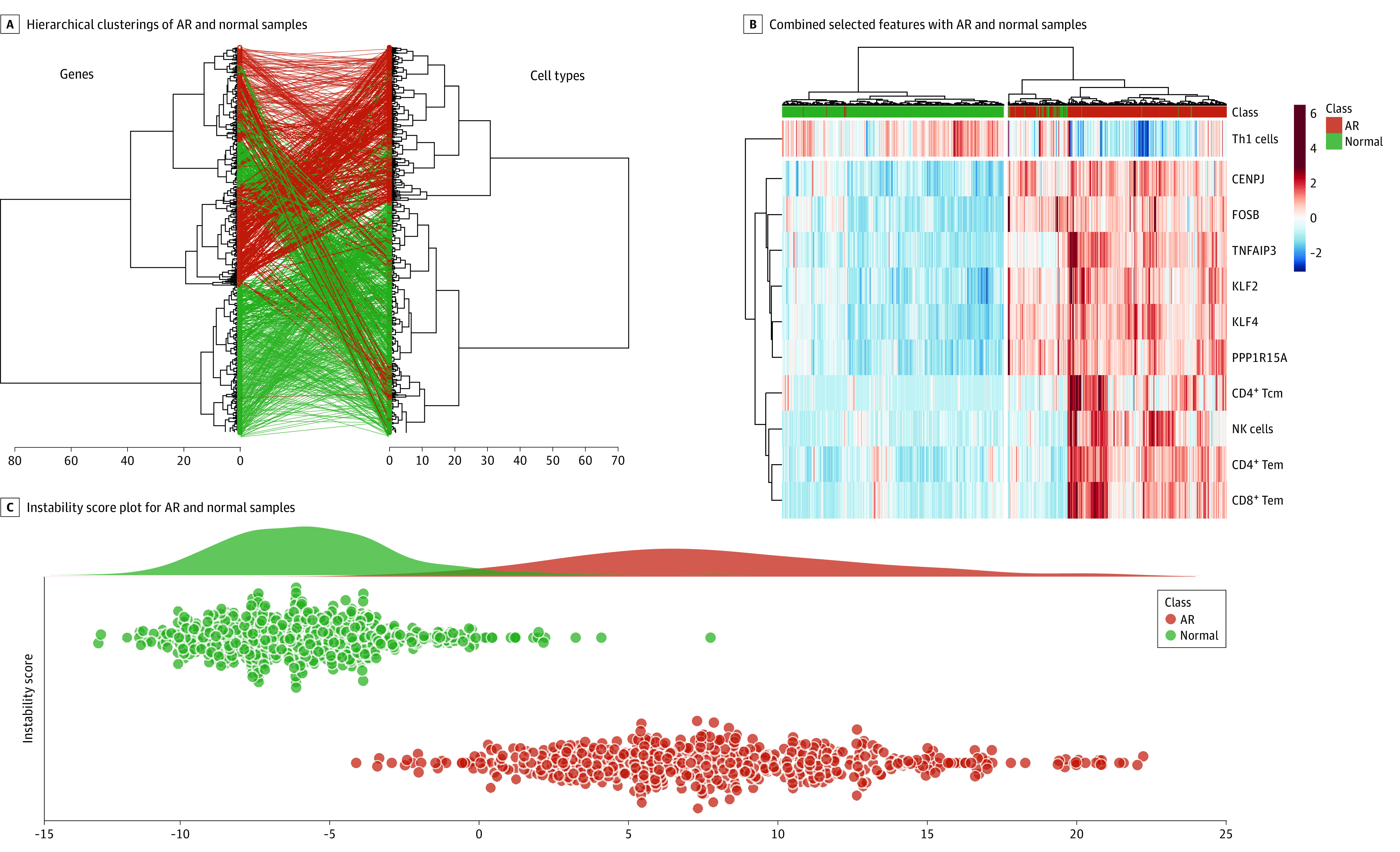
Feature Selected Genes and Cell Types and the Instability Score as Their Combination A, Hierarchical clusterings of acute rejection (AR) and normal samples. B, Combined selected features with AR and normal samples. C, Instability Score plot for AR and normal samples. NK indicates natural killer; Tcm, T-cell central memory; Tem, T-cell effector memory; T_H_1, Type 1 T helper cell.

A generated RF classification model for these 6 genes and 5 cell types, internally validated using 5-fold cross-validation with 100 repeats, obtained an AUROC of 0.98 (sensitivity, 0.94; specificity, 0.94) for the genes alone and an AUROC of 0.92 (sensitivity, 0.85; specificity, 0.88) for the cell types for identification of a tissue sample with histologically confirmed AR (Figure 2A). We further combined the feature selected genes and cell types into 1 score value, called the InstaScore (eMethods in the Supplement), and were able to perform the split into AR and normal samples with a slightly improved AUROC of 0.99, with sensitivity of 0.95 and specificity of 0.94 (Figures 2B and 2C), and an AUCPR of 0.99 (eFigure 7C in the Supplement).

### Selected Features to Create a Scoring Function to Carry Out Precision Subphenotyping of Stable Samples

**Figure 3.**
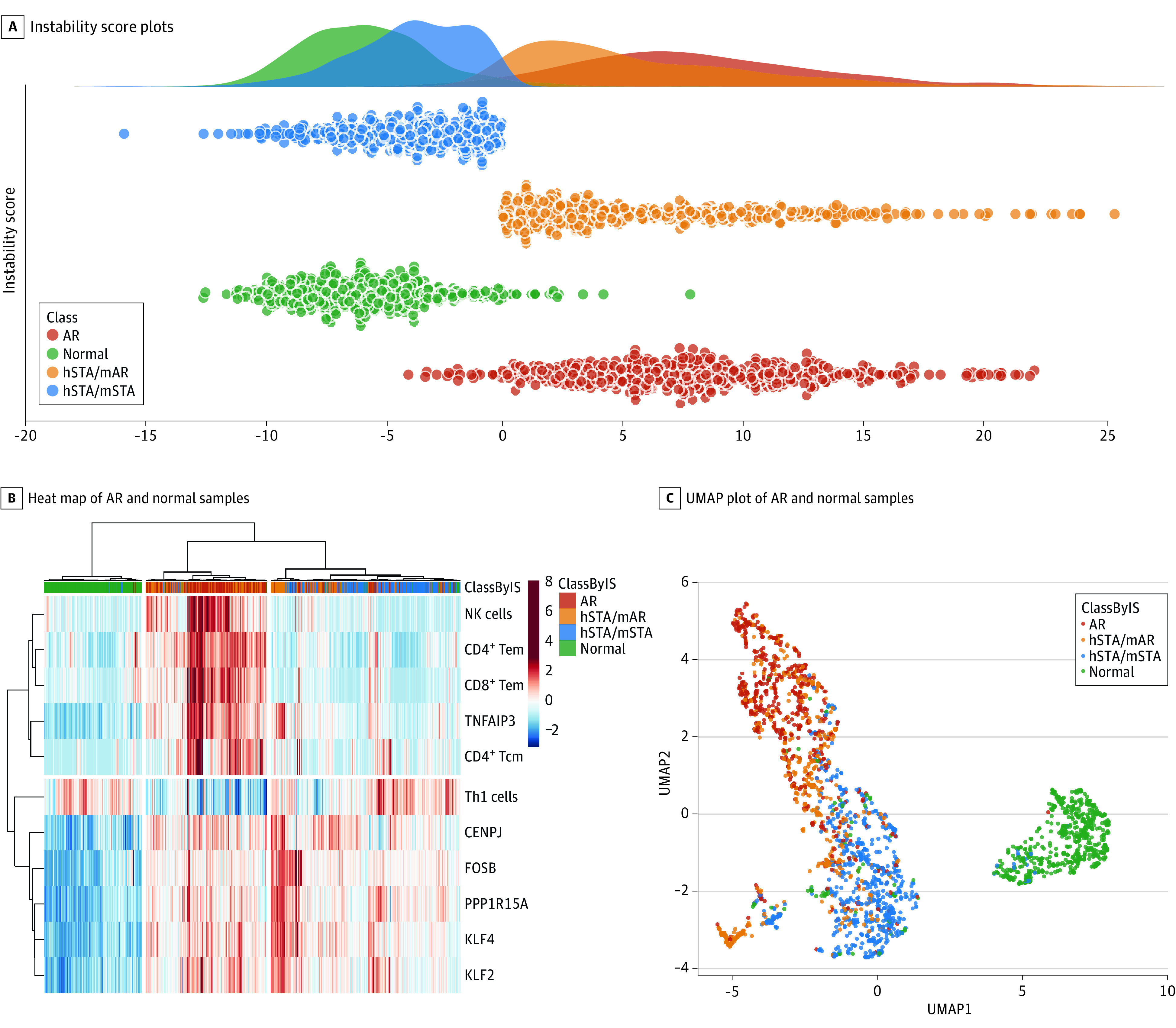
Plots of Acute Rejection (AR), Subphenotyped Histologically Stable (hSTA), and Normal Samples Based on Instability Score Results A, Instability Score plots. B, Heat map of AR and normal samples. C, Uniform Manifold Approximation and Projection (UMAP) plot of AR and normal samples. mAR indicates molecular rejection; mSTA, molecularly stable; NK, natural killer; Tcm, T-cell central memory; Tem, T-cell effector memory; Th1, Type 1 T helper cell.

We then applied the InstaScore to the 1154 transplant samples that were identified by pathologists in each of the data sets as hSTA, classifying samples as more similar to normal kidneys or as more similar to the rejected kidney allograft group (mAR); hSTA/mSTA identified samples with molecular and histologic evidence of no rejection, and hSTA/mAR identified histologically stable allografts with transcriptional evidence of ongoing molecular rejection. The InstaScore identified 528 hSTA grafts (46%) in this study as having mAR (Figure 3A), a misclassification rate in line with previously reported discrepancies in transplant phenotyping across different pathologists.^9,10^

We represented the scores for each sample as a scatterplot in Figures 2C and 3A. The InstaScore was able to significantly distinguish AR and normal samples (1119 samples; *P* < .001; Figure 2C) and distinguish hSTA/mAR and hSTA/mSTA samples (1154 samples; *P* < .001; Figure 3A) by thresholding with zero. The hSTA/mAR samples clustered with AR and separately from hSTA/mSTA samples and had intermediate scores between normal and AR samples (Figure 3B and 3C).

### Validation of hSTA Subphenotyping Using Clinical Follow-up Data

**Figure 4.**
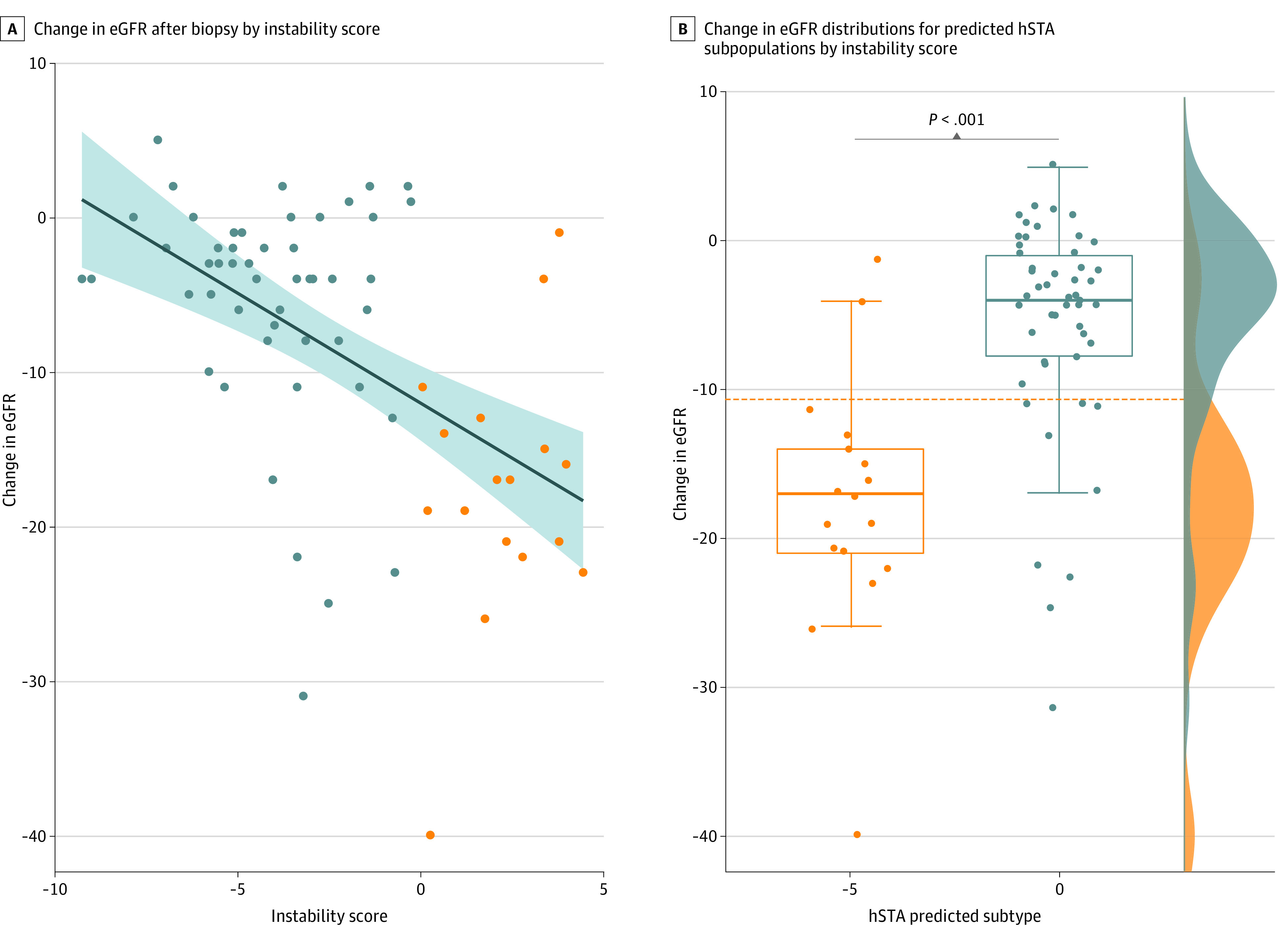
Validation Plots on the Independent Clinical Data A, Change in estimated glomerular filtration rate (eGFR) after biopsy by Instability Score (InstaScore) (*r* = −0.52, *P* < .001). B, Change in eGFR distributions for predicted histologically stable (hSTA) subpopulations by InstaScore (*P* < .001).

In order to assess the functional relevance of the InstaScore by gene expression and cell types, we explored its clinical use in an independent microarray data set from 67 unique patients with hSTA grafts (stable clinical graft function, no donor-specific antibody, and no AR) from a randomized clinical trial^34^ with transcriptional data on serial protocol kidney transplant biopsy samples at 0, 3, 6, 12, and 24 months^35,36^ and with longitudinal functional outcomes up to 5 years after initial engraftment. We tested the correlation association of the locked InstaScore with the change in estimated glomerular filtration rate (eGFR) and graft loss events over this time period and found high correlation values for cell type infiltration and activation model with delta eGFR (Figure 4A) (*r* = 0.52; *P* < .001) and graft loss events (*r* = 0.17; *P* = .26).

Using the predicted hSTA subphenotypes, we estimated a delta eGFR separating threshold of −10 at 5-year follow-up (Figure 4B, *P* < .001). Given these results, it appears that the InstaScore on the 6-month protocol biopsy samples could differentiate patients more likely to have progressive graft injury and decline in graft function over time, even though the 6-month biopsy histology findings, serum creatinine, or donor-specific antibodies cannot provide the same discriminatory information.

## Discussion

Tissue histology is indispensable for the diagnosis of allograft pathology, but its recognized limitations have resulted in the incorporation of data inputs from transcriptional and proteomic studies. Here, we present, to our knowledge, the first unsupervised transcriptional and cell-state framework to map and rephenotype human kidney allografts with undiagnosed graft dysfunction. Unlike other published studies, by others^37,38,39,40,41^ and by members of our group,^2,33,42,43^ that have only focused on general transcriptional perturbations in rejection, the present study is, to our knowledge, the first development and validation of an approach that leverages the statistical power of a large public transcriptional data set. Along with the cell type enrichment analysis using xCell,^28^ we used logistic regression to build the InstaScore. By doing so, we reclassified kidney transplant biopsy samples, otherwise described as hSTA, into samples that have no molecular injury (hSTA/mSTA) and those that are most likely incorrectly annotated as stable but have molecular injury similar to AR (hSTA/mAR).

Approximately half (46%) of the biopsy samples were wrongly annotated as stable and reclassified as hSTA/mAR by the InstaScore; these samples were found to be scattered across multiple data sets, supporting that their presence is not due to failure of histologic characterization at any particular transplant program, and highlighting the failure of histology to detect relevant molecular inflammation. The InstaScore was independently validated for functional relevance,^35^ as it identified hSTA/mAR 6-month protocol biopsy samples that had a higher risk of progressive graft injury and failure at 5 years posttransplant.

The 6 feature selected genes, *KLF4, CENPJ, KLF2, PPP1R15A, FOSB,* and *TNFAIP3,* in the InstaScore are biologically relevant in the immune response and activation and innate immunity. *KLF2, KLF4,* and *TNFAIP3* regulate kidney injury.^44^ *KLF2* is vasoprotective, and *KLF4* is renoprotective; both genes are highly expressed in the endothelium^45,46,47^ and are associated with endothelial ischemia reperfusion injury in AR.^48^ *TNFAIP3* has antiapoptotic and anti-inflammatory functions and expression in endothelial, myeloid, and infiltrating T cells, which results in adverse clinical outcomes in AR.^49,50,51^ *CENPJ* functions as a transcriptional coactivator in STAT 5 signaling and tumor necrosis factor–induced NF-κB–mediated transcription,^52,53^ both of which are central regulators of inflammation. The phosphatase *PPP1R15A* is only expressed in stressed cells and negatively regulates acute kidney injury via type 1 interferon;^54^ clonal expansion; and memory T-cell, plasma cell differentiation, and enhanced B-cell responses.^55^ *FOSB* expression is associated with the progression of kidney disease.^56^ Thus, all InstaScore genes are crucial for endothelial cell integrity, and T-cell activation, and have functional relevance to the kidney and rejection.^57,58^

The 5 feature selected cell types, CD4^+^ Tcm, CD4^+^ Tem, CD8^+^ Tem, NK, and T_H_1, also relate to rejection biology,^59^ together with macrophages, NK cells, and B cells.^60,61,62,63,64^ In the immunologic response to the allograft, T cells terminally differentiate and divide into Tcm cells and CD8^+^ and CD4^+^ Tem cells,^65^ which produce interferon-γ, IL-4, and IL-5 and cytotoxic molecules like granzyme, granulysin, and perforin.^66,67^ CD4^+^ Tcm had the largest effect size in the InstaScore, likely because Tcm cells are characterized by slow effector function and reactive memory and increased response to repeat antigenic stimulation.^68^ In the hSTA/mAR grafts, these cells are primed to differentiate into Tem with low levels of antigen recognition, such as with varying exposure to baseline immunosuppression.^69^ Hence, identification of the hSTA/mAR phenotype in an otherwise clinically and histologically stable allograft may be of critical importance to triage allografts at greater risk of accelerated temporal immune injury.

### Limitations

Given the design of the study, there are a few inherent limitations. First, the publicly available data had limited access to clinical and demographic reports, which could potentially be valuable in incorporation with InstaScore. Second, batch effects had to be controlled for, for which we conducted comparisons of multiple normalizing methods. We chose the RF model to capture possible nonlinear feature interactions to identify the best feature set, although other more complex (eg, neural nets) or less complex (eg, elastic net) methods could also be considered as optional methods. Although the study is based on bulk microarray data, more precise measurement techniques, eg, single-cell RNA sequence, might be used to better capture finer changes in gene expression and cell composition, provide additional validation to the results and, in a future study, refine InstaScore. This may give InstaScore the ability to recognize different types of rejection, which can be identified at the molecular level, long before they can be detected at the histology.

## Conclusions

This prognostic study leverages supervised machine learning on the largest bulk transcriptional human kidney and kidney transplant data set to improve kidney allograft sample phenotyping beyond the capabilities of tissue histology alone. In this study, the InstaScore revealed a level of biologic diversity within the classification of a stable graft not shown by histology alone; based on these findings, the InstaScore may provide an immune map to help refine our understanding of diverse graft functional states. The InstaScore provides a new tool to apply polymerase chain reaction–based analysis of the minimal gene set to kidney allograft biopsy samples embedded in formalin frozen paraffin to identify hSTA/mAR grafts at greater risk for subsequent overt rejection and allograft damage. These patients may benefit from proactive immunosuppression adjustments to reduce molecular inflammation, preserve allograft function, and improve allograft survival.

## Notes

Supplement. **eMethods** **eFigure 1.** Flow Chart **eFigure 2.** Scatterplots of Gene Expression Data After Data Sets Merging **eFigure 3.** PCA Clustering Plot for Differentially Expressed Genes From Analysis of AR vs Normals **eFigure 4.** Pathway Enrichment Analysis of DE Genes **eFigure 5.** Heatmap of Enrichment Scores of Significant Cell Types From the AR vs Normal Comparison **eFigure 6.** Plots of Feature Selected Genes and Cell Types for all AR and Normal Samples **eFigure 7.** AUROC and AUCPR Plots of Feature Selected Genes, Cell Types and InstaScore **eFigure 8.** Combined Benchmark Based on *P*-Value, Delta Statistic and the Percentage of Variability for Batch Correction Methods Tested **eTable 1.** Datasets Collected From Gene Expression Omnibus (GEO) **eTable 2a**. Upregulated Differentially Expressed Genes From SAM Analysis of Comparison of Acute Rejection to Normal Kidney Tissues **eTable 2b**. Downregulated Differentially Expressed Genes From SAM Analysis of Comparison of Acute Rejection to Normal Kidney Tissues **eTable 3.** Cell Types Considered in Cell Type Enrichment Analysis With xCell **eReferences**

## References

[1] Joshee P, Wood AG, Wood ER, Grunfeld EA Meta-analysis of cognitive functioning in patients following kidney transplantation. Nephrol Dial Transplant*.* 2018;33(7):1268-1277. doi:10.1093/ndt/gfx240 28992229 PMC6031036

[2] Naesens M, Khatri P, Li L, Progressive histological damage in renal allografts is associated with expression of innate and adaptive immunity genes. Kidney Int. 2011;80(12):1364-1376. doi:10.1038/ki.2011.245 21881554 PMC4492284

[3] Sigdel TK, Li L, Tran TQ, Non-HLA antibodies to immunogenic epitopes predict the evolution of chronic renal allograft injury. J Am Soc Nephrol. 2012;23(4):750-763. doi:10.1681/ASN.2011060596 22302197

[4] Reeve J, Sellarés J, Mengel M, Molecular diagnosis of T cell-mediated rejection in human kidney transplant biopsies. Am J Transplant. 2013;13(3):645-655. doi:10.1111/ajt.12079 23356949

[5] Sellarés J, Reeve J, Loupy A, Molecular diagnosis of antibody-mediated rejection in human kidney transplants. Am J Transplant. 2013;13(4):971-983. doi:10.1111/ajt.12150 23414212

[6] Halloran PF, Famulski KS, Chang J A Probabilistic approach to histologic diagnosis of antibody-mediated rejection in kidney transplant biopsies. Am J Transplant. 2017;17(1):129-139. doi:10.1111/ajt.13934 27340822

[7] Reeve J, Böhmig GA, Eskandary F, ; MMDx-Kidney study group Assessing rejection-related disease in kidney transplant biopsies based on archetypal analysis of molecular phenotypes. JCI Insight. 2017;2(12):1-14. doi:10.1172/jci.insight.94197 28614805 PMC5470931

[8] Cosio FG, Grande JP, Wadei H, Larson TS, Griffin MD, Stegall MD Predicting subsequent decline in kidney allograft function from early surveillance biopsies. Am J Transplant. 2005;5(10):2464-2472. doi:10.1111/j.1600-6143.2005.01050.x 16162196

[9] Furness PN, Taub N; Convergence of European Renal Transplant Pathology Assessment Procedures (CERTPAP) Project International variation in the interpretation of renal transplant biopsies: report of the CERTPAP Project. Kidney Int. 2001;60(5):1998-2012. doi:10.1046/j.1523-1755.2001.00030.x 11703620

[10] Furness PN, Taub N, Assmann KJM, International variation in histologic grading is large, and persistent feedback does not improve reproducibility. Am J Surg Pathol. 2003;27(6):805-810. doi:10.1097/00000478-200306000-00012 12766585

[11] Haas M, Loupy A, Lefaucheur C, The Banff 2017 kidney meeting report: revised diagnostic criteria for chronic active T cell-mediated rejection, antibody-mediated rejection, and prospects for integrative endpoints for next-generation clinical trials. Am J Transplant. 2018;18(2):293-307. doi:10.1111/ajt.14625 29243394 PMC5817248

[12] Mengel M, Campbell P, Gebel H, Precision diagnostics in transplantation: from bench to bedside. Am J Transplant. 2013;13(3):562-568. doi:10.1111/j.1600-6143.2012.04344.x 23279692

[13] Halloran PF, Famulski KS, Reeve J Molecular assessment of disease states in kidney transplant biopsy samples. Nat Rev Nephrol. 2016;12(9):534-548. doi:10.1038/nrneph.2016.85 27345248

[14] Gulshan V, Peng L, Coram M, Development and validation of a deep learning algorithm for detection of diabetic retinopathy in retinal fundus photographs. JAMA. 2016;316(22):2402-2410. doi:10.1001/jama.2016.17216 27898976

[15] Barrett T, Wilhite SE, Ledoux P, NCBI GEO: archive for functional genomics data sets–update. Nucleic Acids Res. 2013;41(Database issue):D991-D995. doi:10.1093/nar/gks1193 23193258 PMC3531084

[16] Khatri P, Roedder S, Kimura N, A common rejection module (CRM) for acute rejection across multiple organs identifies novel therapeutics for organ transplantation. J Exp Med. 2013;210(11):2205-2221. doi:10.1084/jem.20122709 24127489 PMC3804941

[17] Choi J-W, Kim Y-H, Oh JW Comparative analyses of signature genes in acute rejection and operational tolerance. Immune Netw. 2017;17(4):237-249. doi:10.4110/in.2017.17.4.237 28860953 PMC5577301

[18] Li L, Greene I, Readhead B, Novel therapeutics identification for fibrosis in renal allograft using integrative informatics approach. Sci Rep. 2017;7:39487. doi:10.1038/srep39487 28051114 PMC5209709

[19] Baron D, Ramstein G, Chesneau M, A common gene signature across multiple studies relate biomarkers and functional regulation in tolerance to renal allograft. Kidney Int. 2015;87(5):984-995. doi:10.1038/ki.2014.395 25629549 PMC4424816

[20] R core team. *R: A Language and Environment for Statistical Computing.* R Foundation for Statistical Computing; 2018.

[21] Johnson WE, Li C, Rabinovic A Adjusting batch effects in microarray expression data using empirical Bayes methods. Biostatistics. 2007;8(1):118-127. doi:10.1093/biostatistics/kxj037 16632515

[22] Tusher VG, Tibshirani R, Chu G Significance analysis of microarrays applied to the ionizing radiation response. Proc Natl Acad Sci U S A. 2001;98(9):5116-5121. doi:10.1073/pnas.091062498 11309499 PMC33173

[23] Schwender H. Identifying differentially expressed genes with siggenes. Accessed February 1, 2019. https://www.bioconductor.org/packages/release/bioc/vignettes/siggenes/inst/doc/siggenes.pdf

[24] Goeman JJ, Solari A Multiple hypothesis testing in genomics. Stat Med. 2014;33(11):1946-1978. doi:10.1002/sim.6082 24399688

[25] Reiner-Benaim A FDR control by the BH procedure for two-sided correlated tests with implications to gene expression data analysis. Biom J. 2007;49(1):107-126. doi:10.1002/bimj.200510313 17342953

[26] Yu G, Wang L-G, Han Y, He Q-Y clusterProfiler: an R package for comparing biological themes among gene clusters. OMICS. 2012;16(5):284-287. doi:10.1089/omi.2011.0118 22455463 PMC3339379

[27] Szklarczyk D, Gable AL, Lyon D, STRING v11: protein-protein association networks with increased coverage, supporting functional discovery in genome-wide experimental datasets. Nucleic Acids Res. 2019;47(D1):D607-D613. doi:10.1093/nar/gky1131 30476243 PMC6323986

[28] Aran D, Hu Z, Butte AJ xCell: digitally portraying the tissue cellular heterogeneity landscape. Genome Biol. 2017;18(1):220. doi:10.1186/s13059-017-1349-1 29141660 PMC5688663

[29] Kuhn M. Building predictive models in R using the caret package. J Stat Softw. 2008;28(5):159-160. doi:10.18637/jss.v028.i05

[30] Perez-Riverol Y, Kuhn M, Vizcaíno JA, Hitz M-P, Audain E Accurate and fast feature selection workflow for high-dimensional omics data. PLoS One*.* 2017;12(12):e0189875. doi:10.1371/journal.pone.0189875 29261781 PMC5738110

[31] Github. Kidney_hSTA-subphenotyping. Accessed September 15, 2020. https://github.com/drychkov/Kidney_hSTA_subphenotyping

[32] Kimes PK, Liu Y, Neil Hayes D, Marron JS Statistical significance for hierarchical clustering. Biometrics. 2017;73(3):811-821. doi:10.1111/biom.12647 28099990 PMC5708128

[33] Sarwal M, Chua M-S, Kambham N, Molecular heterogeneity in acute renal allograft rejection identified by DNA microarray profiling. N Engl J Med. 2003;349(2):125-138. doi:10.1056/NEJMoa035588 12853585

[34] Steroid-free versus steroid-based immunosuppression in pediatric renal (kidney) transplantation. Clinicaltrials.gov identifier: NCT00141037. Updated November 29, 2016 Accessed November 18, 2018. https://clinicaltrials.gov/ct2/show/NCT00141037

[35] Sarwal MM, Ettenger RB, Dharnidharka V, Complete steroid avoidance is effective and safe in children with renal transplants: a multicenter randomized trial with three-year follow-up. Am J Transplant. 2012;12(10):2719-2729. doi:10.1111/j.1600-6143.2012.04145.x 22694755 PMC3681527

[36] Naesens M, Salvatierra O, Benfield M, ; SNS01-NIH-CCTPT Multicenter Trial Subclinical inflammation and chronic renal allograft injury in a randomized trial on steroid avoidance in pediatric kidney transplantation. Am J Transplant. 2012;12(10):2730-2743. doi:10.1111/j.1600-6143.2012.04144.x 22694733 PMC3459071

[37] Cippà PE, Liu J, Sun B, Kumar S, Naesens M, McMahon AP A late B lymphocyte action in dysfunctional tissue repair following kidney injury and transplantation. Nat Commun. 2019;10(1):1157. doi:10.1038/s41467-019-09092-2 30858375 PMC6411919

[38] Dorr CR, Oetting WS, Jacobson PA, Israni AK Genetics of acute rejection after kidney transplantation. Transpl Int. 2018;31(3):263-277. doi:10.1111/tri.13084 29030886 PMC6260834

[39] Edemir B, Reuter S, Borgulya R, Acute rejection modulates gene expression in the collecting duct. J Am Soc Nephrol. 2008;19(3):538-546. doi:10.1681/ASN.2007040513 18216318 PMC2391056

[40] Hernandez-Fuentes M, Christakoudi S, Runglall M, A signature of gene expression in peripheral blood that enables earlier detection of acute rejection in kidney transplant recipients. Transplantation. 2018;102:S180. doi:10.1097/01.tp.0000542822.75680.9c

[41] Spivey TL, Uccellini L, Ascierto ML, Gene expression profiling in acute allograft rejection: challenging the immunologic constant of rejection hypothesis. J Transl Med. 2011;9(1):174. doi:10.1186/1479-5876-9-174 21992116 PMC3213224

[42] Pineda S, Sigdel TK, Chen J, Jackson AM, Sirota M, Sarwal MM Novel non-histocompatibility antigen mismatched variants improve the ability to predict antibody-mediated rejection risk in kidney transplant. Front Immunol. 2017;8(JAN):1687. doi:10.3389/fimmu.2017.01687 29259604 PMC5723302

[43] Sigdel TK, Bestard O, Tran TQ, A computational gene expression score for predicting immune injury in renal allografts. PloS One*.* 2015;10(9):e0138133. doi:10.1371/journal.pone.0138133 PMC4569485 26367000

[44] Rane MJ, Zhao Y, Cai L Krϋppel-like factors (KLFs) in renal physiology and disease. EBioMedicine. 2019;40:743-750. doi:10.1016/j.ebiom.2019.01.021 30662001 PMC6414320

[45] Boon RA, Leyen TA, Fontijn RD, KLF2-induced actin shear fibers control both alignment to flow and JNK signaling in vascular endothelium. Blood. 2010;115(12):2533-2542. doi:10.1182/blood-2009-06-228726 20032497

[46] Ke B, Zhang A, Wu X, Fang X The role of Krüppel-like factor 4 in renal fibrosis. Front Physiol. 2015;6(NOV):327. doi:10.3389/fphys.2015.00327 26617530 PMC4641914

[47] Sangwung P, Zhou G, Nayak L, KLF2 and KLF4 control endothelial identity and vascular integrity. JCI Insight. 2017;2(4):e91700. doi:10.1172/jci.insight.91700 28239661 PMC5313061

[48] Mallipattu SK, Estrada CC, He JC The critical role of Krüppel-like factors in kidney disease. Am J Physiol Renal Physiol. 2017;312(2):F259-F265. doi:10.1152/ajprenal.00550.2016 27852611 PMC5336586

[49] Luft FC Zinc fingers protect the kidney from ischemia/reperfusion injury. J Mol Med (Berl). 2008;86(12):1297-1300. doi:10.1007/s00109-008-0411-6 18941730

[50] Lutz J, Luong A, Strobl M, The A20 gene protects kidneys from ischaemia/reperfusion injury by suppressing pro-inflammatory activation. J Mol Med (Berl). 2008;86(12):1329-1339. doi:10.1007/s00109-008-0405-4 18813897

[51] Avihingsanon Y, Ma N, Csizmadia E, Expression of protective genes in human renal allografts: a regulatory response to injury associated with graft rejection. Transplantation. 2002;73(7):1079-1085. doi:10.1097/00007890-200204150-00011 11965035

[52] Koyanagi M, Hijikata M, Watashi K, Masui O, Shimotohno K Centrosomal P4.1-associated protein is a new member of transcriptional coactivators for nuclear factor-kappaB. J Biol Chem. 2005;280(13):12430-12437. doi:10.1074/jbc.M410420200 15687488

[53] Peng B, Sutherland KD, Sum EYM, CPAP is a novel stat5-interacting cofactor that augments stat5-mediated transcriptional activity. Mol Endocrinol. 2002;16(9):2019-2033. doi:10.1210/me.2002-0108 12198240

[54] Plazy C, Dumestre-Pérard C, Sarrot-Reynauld F, Letter to the editor: protein phosphatase 1 subunit Ppp1r15a/GADD34 is overexpressed in systemic lupus erythematosus and related to the expression of type I interferon response genes. Autoimmun Rev*.* 2019;18(2):211-213. doi:10.1016/j.autrev.2018.09.007 30578961

[55] Kamphuis E, Junt T, Waibler Z, Forster R, Kalinke U Type I interferons directly regulate lymphocyte recirculation and cause transient blood lymphopenia. Blood. 2006;108(10):3253-3261. doi:10.1182/blood-2006-06-027599 16868248

[56] Park HJ, Kim JW, Cho BS, Chung JH Association of FOS-like antigen 1 promoter polymorphism with podocyte foot process effacement in immunoglobulin A nephropathy patients. J Clin Lab Anal. 2014;28(5):391-397. doi:10.1002/jcla.21699 24652774 PMC6807477

[57] Lin Y, Lewallen EA, Camilleri ET, RNA-seq analysis of clinical-grade osteochondral allografts reveals activation of early response genes. J Orthop Res. 2016;34(11):1950-1959. doi:10.1002/jor.23209 26909883 PMC4993686

[58] Mutoh J, Ohsawa M, Hisa H Effect of naloxone on ischemic acute kidney injury in the mouse. Neuropharmacology. 2013;71:10-18. doi:10.1016/j.neuropharm.2013.03.001 23523991

[59] Farrar CA, Kupiec-Weglinski JW, Sacks SH The innate immune system and transplantation. Cold Spring Harb Perspect Med. 2013;3(10):a015479-a015479. doi:10.1101/cshperspect.a015479 24086066 PMC3784815

[60] Imig JD, Ryan MJ. Immune and inflammatory role in renal disease. Compr Physiol. 2013;3(2):957-976. 23720336 10.1002/cphy.c120028 PMC3803162

[61] Weller S, Varrier M, Ostermann M Lymphocyte function in human acute kidney injury. Nephron. 2017;137(4):287-293. doi:10.1159/000478538 28662513

[62] Ingulli E Mechanism of cellular rejection in transplantation. Pediatr Nephrol. 2010;25(1):61-74. doi:10.1007/s00467-008-1020-x 21476231 PMC2778785

[63] Yazdani S, Callemeyn J, Gazut S, Natural killer cell infiltration is discriminative for antibody-mediated rejection and predicts outcome after kidney transplantation. Kidney Int. 2019;95(1):188-198. doi:10.1016/j.kint.2018.08.027 30396694

[64] Crome SQ, Lang PA, Lang KS, Ohashi PS Natural killer cells regulate diverse T cell responses. Trends Immunol. 2013;34(7):342-349. doi:10.1016/j.it.2013.03.002 23601842

[65] Sallusto F, Geginat J, Lanzavecchia A Central memory and effector memory T cell subsets: function, generation, and maintenance. Annu Rev Immunol. 2004;22(1):745-763. doi:10.1146/annurev.immunol.22.012703.104702 15032595

[66] Opata MM, Stephens R Early decision: effector and effector memory T cell differentiation in chronic infection. Curr Immunol Rev. 2013;9(3):190-206. doi:10.2174/1573395509666131126231209 24790593 PMC4000274

[67] Sarwal MM, Jani A, Chang S, Granulysin expression is a marker for acute rejection and steroid resistance in human renal transplantation. Hum Immunol. 2001;62(1):21-31. doi:10.1016/S0198-8859(00)00228-7 11165712

[68] Pepper M, Jenkins MK Origins of CD4(+) effector and central memory T cells. Nat Immunol. 2011;12(6):467-471. doi:10.1038/ni.2038 21739668 PMC4212218

[69] Segundo DS, Fernández-Fresnedo G, Gago M, Changes in the number of circulating T_CM_ and T_EM_ subsets in renal transplantation: relationship with acute rejection and induction therapy. Kidney Int Suppl (2011). 2011;1(2):31-35. doi:10.1038/kisup.2011.9 25018900 PMC4089667

