# Supplementary material for: Molecular Diversity of Clinically Stable Human Kidney Allografts
Source: JAMA Netw Open. 2021 Jan 25;4(1):e2035048. doi: 10.1001/jamanetworkopen.2020.35048 (PMC7835722; doi:10.1001/jamanetworkopen.2020.35048)
Supplement: Supplement. — eMethods eFigure 1. Flow Chart eFigure 2. Scatterplots of Gene Expression Data After Data Sets Merging eFigure 3. PCA Clustering Plot for Differentially Expressed Genes From Analysis of AR vs Normals eFigure 4. Pathway Enrichment Analysis of DE Genes eFigure 5. Heatmap of Enrichment Scores of Significant Cell Types From the AR vs Normal Comparison eFigure 6. Plots of Feature Selected Genes and Cell Types for all AR and Normal Samples eFigure 7. AUROC and AUCPR Plots of Feature Selected Genes, Cell Types and InstaScore eFigure 8. Combined Benchmark Based on P-Value, Delta Statistic and the Percentage of Variability for Batch Correction Methods Tested eTable 1. Datasets Collected From Gene Expression Omnibus (GEO) eTable 2a. Upregulated Differentially Expressed Genes From SAM Analysis of Comparison of Acute Rejection to Normal Kidney Tissues eTable 2b. Downregulated Differentially Expressed Genes From SAM Analysis of Comparison of Acute Rejection to Normal Kidney Tissues eTable 3. Cell Types Considered in Cell Type Enrichment Analysis With xCell eReferences [file jamanetwopen-e2035048-s001.pdf]

Supplementary Online Content

Rychkov D, Sur S, Sirota M, Sarwal MM. Molecular diversity of clinically stable human kidney allografts. *JAMA Netw Open*. 2021;4(1):e2035048. doi:10.1001/jamanetworkopen.2020.35048

eMethods

eFigure 1. Flow Chart

eFigure 2. Scatterplots of Gene Expression Data After Data Sets Merging

eFigure 3. PCA Clustering Plot for Differentially Expressed Genes From Analysis of AR vs Normals

eFigure 4. Pathway Enrichment Analysis of DE Genes

eFigure 5. Heatmap of Enrichment Scores of Significant Cell Types From the AR vs Normal Comparison

eFigure 6. Plots of Feature Selected Genes and Cell Types for all AR and Normal Samples

eFigure 7. AUROC and AUCPR Plots of Feature Selected Genes, Cell Types and InstaScore

eFigure 8. Combined Benchmark Based on *P*-Value, Delta Statistic and the Percentage of Variability for Batch Correction Methods Tested

eTable 1. Datasets Collected From Gene Expression Omnibus (GEO)

eTable 2a. Upregulated Differentially Expressed Genes From SAM Analysis of Comparison of Acute Rejection to Normal Kidney Tissues

eTable 2b. Downregulated Differentially Expressed Genes From SAM Analysis of Comparison of Acute Rejection to Normal Kidney Tissues

eTable 3. Cell Types Considered in Cell Type Enrichment Analysis With xCell

eReferences

This supplementary material has been provided by the authors to give readers additional information about their work.

© 2021 Rychkov D et al. *JAMA* Network Open.

eMethods

Data collection. We carried out a comprehensive search for publicly available microarray data at NCBI GEO (Gene Expression Omnibus) database [1] (http://www.ncbi.nlm.nih.gov/geo/). We used the keywords "kidney", "renal", "transplant", and "biopsy", among organisms "Homo Sapiens" and study type "Expression profiling by array". We identified and collected publicly available microarray data from 38 datasets with a total of 4,845 human kidney biopsy samples (eTable 1). For each dataset, we carefully collected the platform information, annotations, and expression data. The gene expression measurements were made on 18 different platforms (eTable 1). The commercial chips were designed by the manufacturers Affymetrix, Agilent and Illumina, and the custom chips were by Functional Genomics Facility at Stanford University and Bioinformatics and Gene Network Research Group at Zhejiang University. The number of probes varied widely from 1,347 to 54,675. In order to preserve as many genes as possible for further analysis we filtered out two datasets GSE26578 and GSE1563 with the lowest number of probes. We also filtered out 5 more datasets: GSE1743, GSE21785, GSE98320, GSE83486, and GSE109346, due to multiple absent or poor annotations such that it was impossible to confidently identify phenotypes associated with the individual samples. The dataset GSE343 was removed since the data of only log2 ratios of intensities in Cy5 and Cy3 channels were available. We found two datasets, GSE14700 and GSE82337, and one sample from GSE36059 with very sparse expression data and significant percentage of missing values, hence we filtered out these datasets as well. In the last step, we examined all datasets for any duplicate samples since many studies recycle previously published data. We were able to identify and remove 179 duplicate samples from GSE11166, GSE14328, GSE34437, GSE50058, and GSE72925 based on available metadata. We then used an R package DoppelgangR [2] that is based on calculations of correlations between samples and manually curated its results for any false positives and identified 257 samples as highly possible duplicates that were then also removed. Using the PCA plots, boxplots, and density plots, we analyzed the datasets for any presence of outliers. After stringent data quality control procedures, we composed the final dataset consisted of 28 studies with 2,273 samples. Their diagnostic annotations included 510 Acute rejection (AR) (including Antibody mediated rejection (ABMR), T cell mediated rejection (TCMR), AR, AR with Chronic allograft nephropathy (AR+CAN), Borderline rejection (BL), BL+CAN, Mixed rejection), 1,154 Stable (STA), and 609 Normal (i.e., biopsy taken prior to transplantation). The summary for the collected studies is represented in eTable 1.

Data processing and normalization. Several pre-processing steps were applied prior to the main analysis. Raw fluorescence intensity data stored in .CEL or .txt files were downloaded and pre-processed depending on the platform. The data processing included background correction, log2 transformation, quantile normalization and probe to gene mapping using R language version 3.5.1 [3]. For the Affymetrix platform, we used the R package *SCAN.UPC* [4] available at Bioconductor [5] (http://ww.bioconductor.org). In contrast to some other popular multi-array normalization algorithms like RMA [6] that estimate probe-level effects and standardize variances across arrays based on the information from a whole dataset, SCAN.UPC is a single-array method that normalizes every sample independently from other samples. This is considered as an advantage [4] since this approach is robust to any influence from possible outliers in the data. The database for the mapping between probes and Entrez gene IDs were taken from the BrainArray resource [7] version 22 (http://brainarray.mbni.med.umich.edu/Brainarray/Database/CustomCDF/22.0.0/entrezg.asp). For the Agilent and Illumina platforms, we downloaded non-normalized raw data and performed data processing using *neqc()* function within *limma* package [8] from Bioconductor. This algorithm [9] estimates parameters based on normal-exponential (normexp) convolutional model with joint likelihood estimation and with the help of negative control probes. The offset 16 was added to the intensities after the background adjustment by default, as it was shown as the most optimal value to improve FDR of the normexp algorithm [8]. However, some data from the Agilent and Stanford platforms did not contain any negative control probes. Therefore, we used similar processing steps manually to reproduce the methodology by applying *backgroundCorrect()* function from *limma* package by the *mle normexp*

© 2021 Rychkov D et al. *JAMA* Network Open.

method with offset *16*. *Log2* transformation and quantile normalization was performed after this. The probe-gene mapping was implemented using the information from *biomaRt* database [10] or GPL files.

To gain additional statistical power from the large dataset we were able to collect, we chose to merge all the studies to perform a meta-analysis. In order to merge data from different studies and different platforms we had to first correct for potential batch effects. There are a number of papers that address this issue [11–15], but unfortunately, a one-fits-all solution doesn't exist. Different normalization methods have their advantages and disadvantages [16–18] in removing batch effects, however, they can also become a critical problem in correcting the imbalanced data [11,15]. We examined the performance of several approaches that included *ComBat* [19] as a part of sva package [20], *Quantile Normalization (QN)* [21], *Remove Unwanted Variation (RUV)* [22] and *Harman* [23]. We identified superior batch effect minimization with *ComBat* in comparison to other methods and used it to normalize the data. The process of data aggregation, normalization and merging is schematically described in eFigure 1a.

Cross-study normalization in data merging. We have examined several batch correction methods: *ComBat* [19] as a part of sva package [20], *Quantile Normalization (QN)* [21], *Remove Unwanted Variation (RUV)* [22] and *Harman* [23]. Currently, the most common technique to remove the systematic batch effects from biological data is *ComBat* [19] which based on empirical Bayes method to estimate batch effects and to adjust data across genes. However, often a major limitation of its use is missed: the studies merged have to be more or less balanced with respect to the case-control breakdown of samples. If it is not the case, some biological heterogeneity can vanish [15]. The breakdown by phenotype for each study we use in our work is represented in the eTable 1 and shows presence of some imbalance in outcomes. Therefore, we wanted to additionally examine some other normalization methods comprehensively.

The *Quantile Normalization* technique is the same method used for normalization at the probe level, but as it was shown before [21] it can be applied for batch effect elimination as well.

The *RUV* method [22] is based on adjustment on so-called negative control genes, that are expected not to be differentially expressed across phenotypes. There is still an open question which set of genes would be most suitable for this role. Basically, so far there are two possible ways to obtain a list of control genes: housekeeping genes and empirically found ones. The housekeeping genes are defined to be expressed on the similar level across all tissues and involved in basic cell processes [24]. However, besides the fact that there is no fully established list of housekeeping genes [25], it was shown that they might not be the best to represent negative controls in adjustments [22] since they can be differentially expressed in some tissues [12] or related to diseases [26]. Another method is to empirically find genes that are expressed steadily across merging studies. However, there is some freedom in setting a number of such genes. Too small number can be not enough for proper adjustment, too large – can include some genes important for a current study. Some dependence on studies involved is presented in the search for negative control genes. For some discussion of advantages and disadvantages of both methods see reference [27]. Another challenging factor in applying the RUV method is the parameters adjustment. Depending on implementation of the algorithm, there are two main parameters to adjust: the dimension of the unwanted systematic noise and the ridge smoothing parameter. To find an optimal set of parameters is actually a tricky problem. In our study, we examined three different implementations of RUV method in R packages RUVcorr [27], RUVnormalize [28], and RUVSeq [29] (RUVg function). The first two packages implement naïve RUV-random method which is a variation of the RUV-2 method originally described in [22]. The third method RUVg within RUVseq package is originally designed as a discrete version of RUV-2 methods for RNA-seq counts data. We used this method for performance comparison with other approaches. We compared the performance of these RUV implementations with the housekeeping and empirical negative control genes. We also varied the parameter of the noise dimension *k* but set the smoothing ridge parameter to 0.001 for RUVnormalize and 0 for RUVcorr.

© 2021 Rychkov D et al. *JAMA* Network Open.

Another promising normalization method we examined is *Harman* [23]. This method is a Principal Component Analysis based optimization technique that maximizes batch removal but keeps some probability of the overcorrection as a parameter. We compared the method performance with the overcorrection parameter set to 0.95.

We considered three types of benchmarks to perform the comparison for normalization methods. We computed the percentage of variability in first ten principal components that can be explained by batch (i.e. by dataset study) and kept the maximum value among those ten as one of three benchmarks. For the other two we used the R package gPCA [30] to compute guided principal components (i.e. the principal components of batch modified data) and obtained a p-value (i.e. the probability of having batch effect in data) and delta statistic (i.e. the ratio of guided and unguided first principal component; the lower the better). Since all three metrics are in the range from 0 to 1, we sumed them and normalize to one and used as a final metric to justify the normalization method performance. The results are represented in the eFigure 7.

We found the Harman normalization to outperform all methods followed by ComBat and RUV implementations in RUVcorr and RUVseq with housekeeping genes at the noise dimension parameter k = 15-17. Further comparison analysis showed that while the data seems batch free, RUV and Harman adjusted a bit too much leaving minimum of biological variability, resulting in much fewer differentially expressed genes in comparison to ComBat. Therefore, we decided to use ComBat for cross-study normalization to keep as much heterogeneity as possible while successfully adjusting for batch effects. We observed that the results of the normalization performance vary depending on datasets: their number, platform, processing methods. There is no one-fit-all technique that should be used blindly to correct for batch effects when merging data. Therefore, it should be advised to perform such comparisons of normalization methods each time performing meta-analysis to choose best among available methods [14].

Statistics. To identify differentially expressed genes in the first analysis AR vs Normal we used the Significance Analysis of Microarrays (SAM) [31] method that was implemented in the R package *siggenes* [32]. We utilized the false discovery rate (FDR) [33] with Benjamini-Hochberg procedure [34] for multiple testing correction and use the adjusted cutoff of 0.05. For the second level of significance, we selected only those genes that have the fold change greater than 1.5.

Pathway Analysis. We leveraged the Gene Ontology database using the gene set enrichment analysis implemented in the R package *clusterProfiler* [35] to perform functional annotations for the significantly up- and down-regulated genes. We used FDR multiple correction method with the enrichment significance cut-off at level 0.05. For the gene network analysis, we utilized the STRING protein–protein association networks database [36] (https://string-db.org).

Cell type enrichment analysis. In order to estimate the presence of certain cell types in biopsy samples, we leveraged a recently published cell type enrichment tool *xCell* [37]. xCell leverages gene expression data from microarray and RNA-seq experiments and is used to perform enrichment analysis for up to 64 immune and stromal cell types. We focused on 34 immune related and 11 non-immune cell types (eTable 3) that we selected manually as relevant to the transplant injury process. In our analysis, we used a dedicated R package that is available on the author's GitHub account for this purpose. This analytical method is a gene signatures-based method and converts the gene expression into cell type enrichment scores. The authors especially emphasize that this is not a deconvolution method that provides percentage of cell types containing in a tissue but rather the enrichment tool allowing to compare samples

© 2021 Rychkov D et al. *JAMA* Network Open.

for each cell type but not otherwise. The enrichment scores for each cell type were used to compare AR and Normal samples and to identify cell types that are significantly different in individuals with AR as opposed to normal controls by performing the non-parametric two-sample Mann-Whitney-Wilcoxon statistical test. We utilized the multiple testing correction by using the Benjamini-Hochberg method. Adjusted p-value < 0.05 was used as the threshold.

Feature selection procedure. In our efforts to select the most important features in distinguishing AR vs Normal samples, we performed the following steps. First, we split the whole data into training and testing sets in the ratio 80:20 and performed all feature selection procedures on the training set with benchmarking on the testing set. After identifying the significant features from a statistical test described above on the training set, we searched for features that correlate with the outcome no less than 2/3 of a maximum correlation value. In the final step, we applied Recursive Feature Elimination (RFE) technique with Random Forest (RF) model from the R package *caret* [38]. We used 5-fold cross validation (CV) technique with 100 repeats. To benchmark the model, we used the area under the ROC curve that is more suitable for data with some disbalance in outcome - in our case the ratio AR:Normal is 0.84. To decrease the bias of random split as well as to avoid the model overfitting, we also introduced the tolerance of 1% to the feature selection mechanism, i.e. the algorithm was choosing the simplest model with the smallest number of features that performs within range 99-100% of the best model.

To perform the steps described above, we adopted the R package *feseR* [39] and modified it to implement the parallel computations, the AUROC metric for model benchmarking, and the tolerance parameter of model performance. After the feature selection steps, we benchmarked the features with RF model on the testing set.

Instability Score and hSTA sub-phenotyping. The method of sub-phenotyping hSTA samples is formed on creating a scoring system based on selected features and scoring the hSTA samples. Based on the score values, histologically STA samples are identified as *molecularly* AR or *molecularly* STA. We denoted this split as hSTA/mAR and hSTA/mSTA, respectively.

For our current analysis, two types of data were available: gene expression data and cell type enrichment scores (obtained computationally using xCell). Based on these two data types, we performed the feature selection procedure described above to find sets of genes and cell types that highly associated with AR. Next, we z-scaled each feature, a gene or a cell type, and built a logistic regression model with all features to identify feature importance as model coefficients. Using these coefficients, we created a linear formula to compute a score, that we called the *Instability Score* (or *InstaScore*):

$$\begin{aligned} InstaScore = {} & 0.596 + 2.096 \times \mathrm{KLF4} + 2.534 \times \mathrm{CENPJ} + 0.311 \times \mathrm{KLF2} + 1.447 \times \mathrm{PPP1R15A} \\ & - 1.633 \times \mathrm{FOSB} + 0.268 \times \mathrm{TNFAIP3} + 2.249 \times \mathrm{NK\ cells} + 0.542 \times \mathrm{CD4{+}\ T_{cm}\ cells} \\ & + 0.833 \times \mathrm{CD4{+}\ T_{em}\ cells} + 0.709 \times \mathrm{CD8{+}\ T_{em}\ cells} + 0.146 \times \mathrm{Th_1\ cells}. \end{aligned}$$

The positive *InstaScore* values separate AR from Normal samples which obtain negative values. By following the same steps, we then computed the *InstaScore* for the histologically STA samples and applied the zeroth threshold to obtain the split into mAR and mSTA subtypes. This whole approach is schematically represented in the form of the flow chart in the eFigures 1b and 1c.

© 2021 Rychkov D et al. *JAMA* Network Open.

eFigure 1. Flow Chart

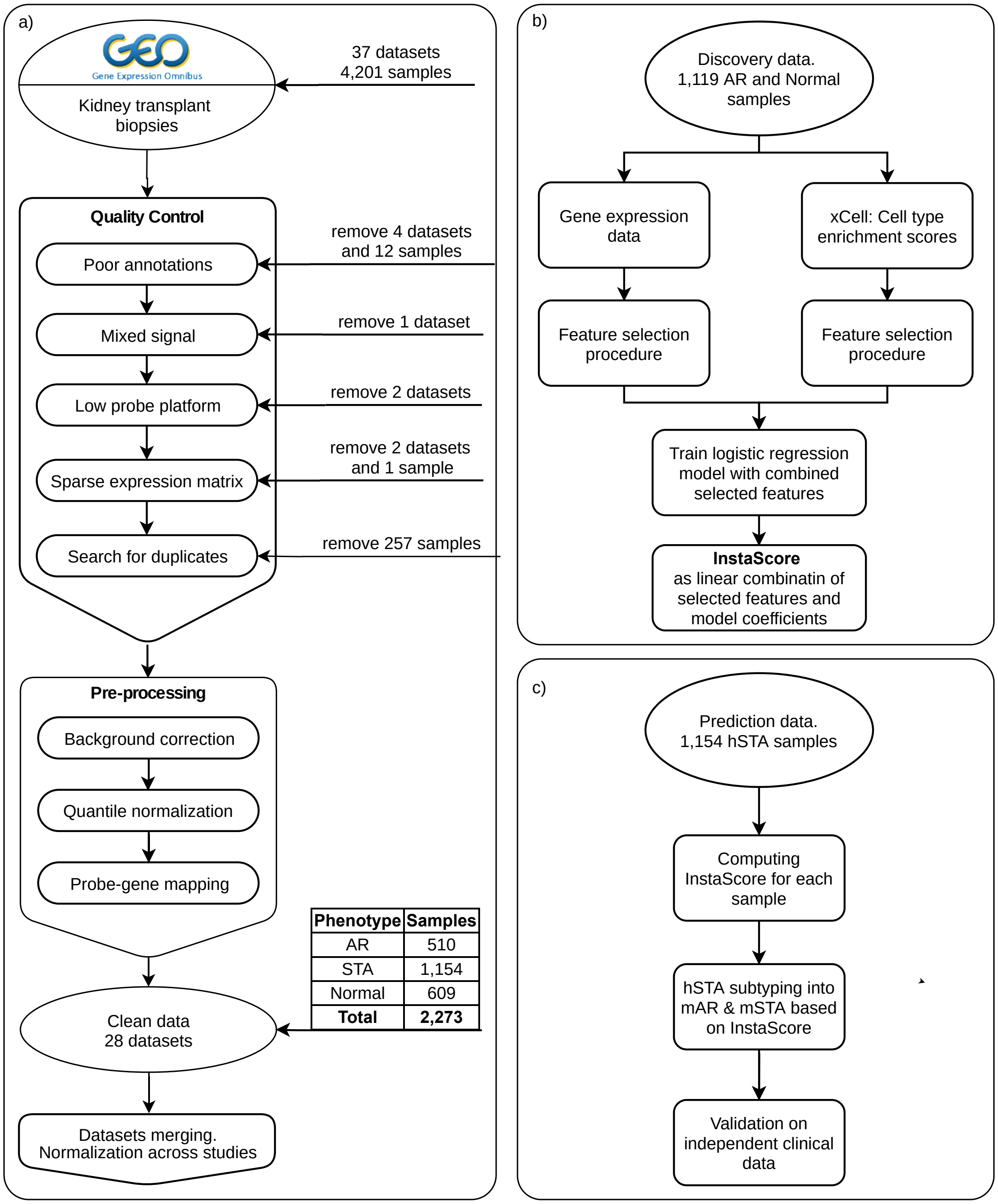

(a) Data pre-processing and Normalization. (b) Feature selection and creating the Instability Score (*InstaScore*). (c) Applying *InstaScore* to hSTA for sub-phenotyping and validating results.

© 2021 Rychkov D et al. *JAMA* Network Open.

eFigure 2. Scatterplots of Gene Expression Data After Data Sets Merging

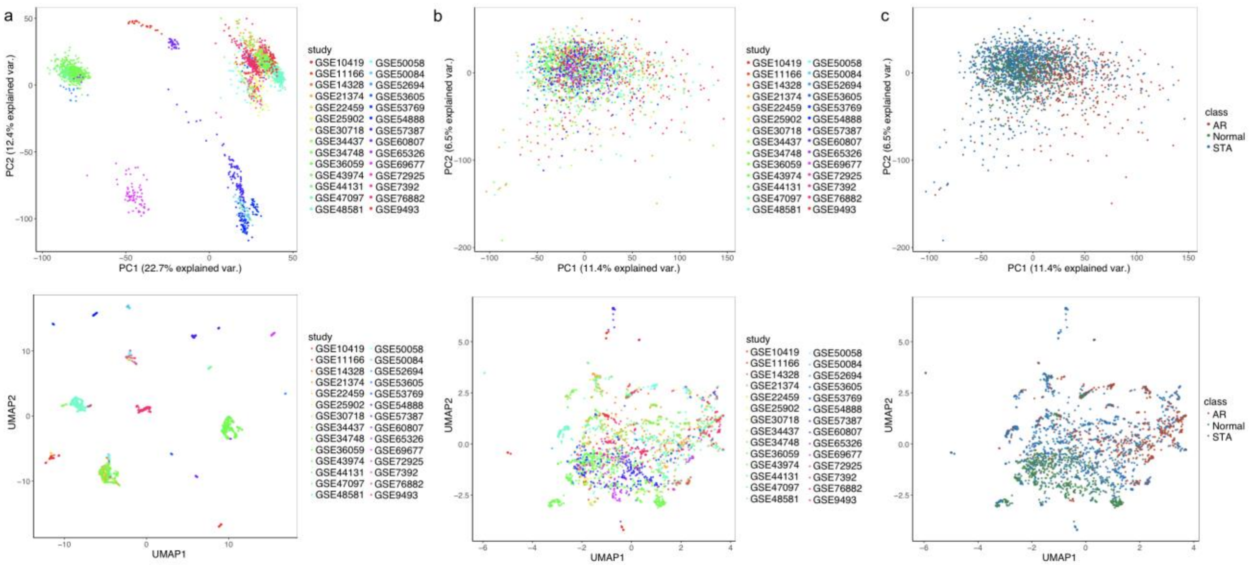

PCA and UMAP plots (a) before normalization colored by study, (b) after normalization with ComBat colored by study, (c) after normalization colored by phenotypes.

© 2021 Rychkov D et al. *JAMA* Network Open.

eFigure 3. PCA Clustering Plot for Differentially Expressed Genes From Analysis of AR vs Normals

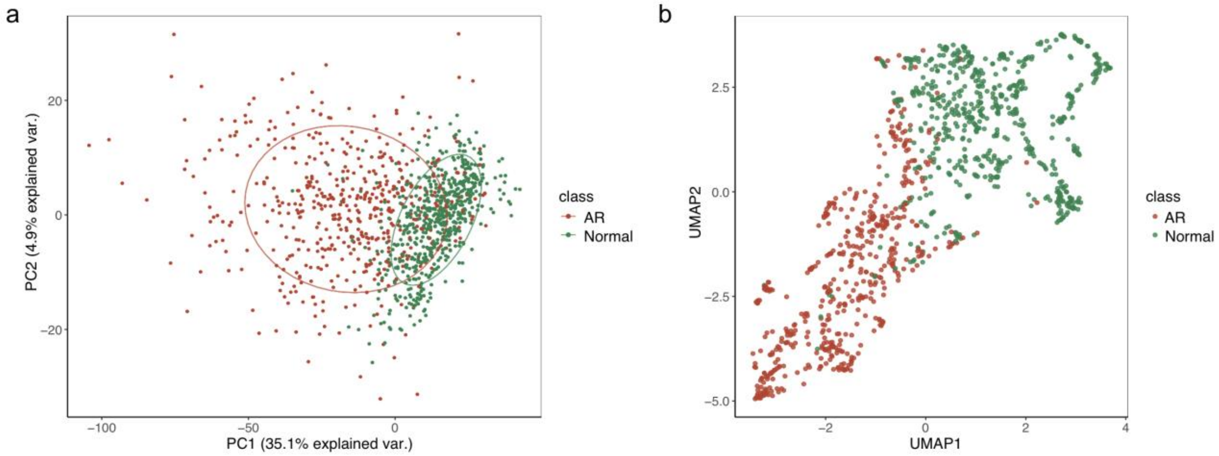

© 2021 Rychkov D et al. *JAMA* Network Open.

eFigure 4. Pathway Enrichment Analysis of DE Genes

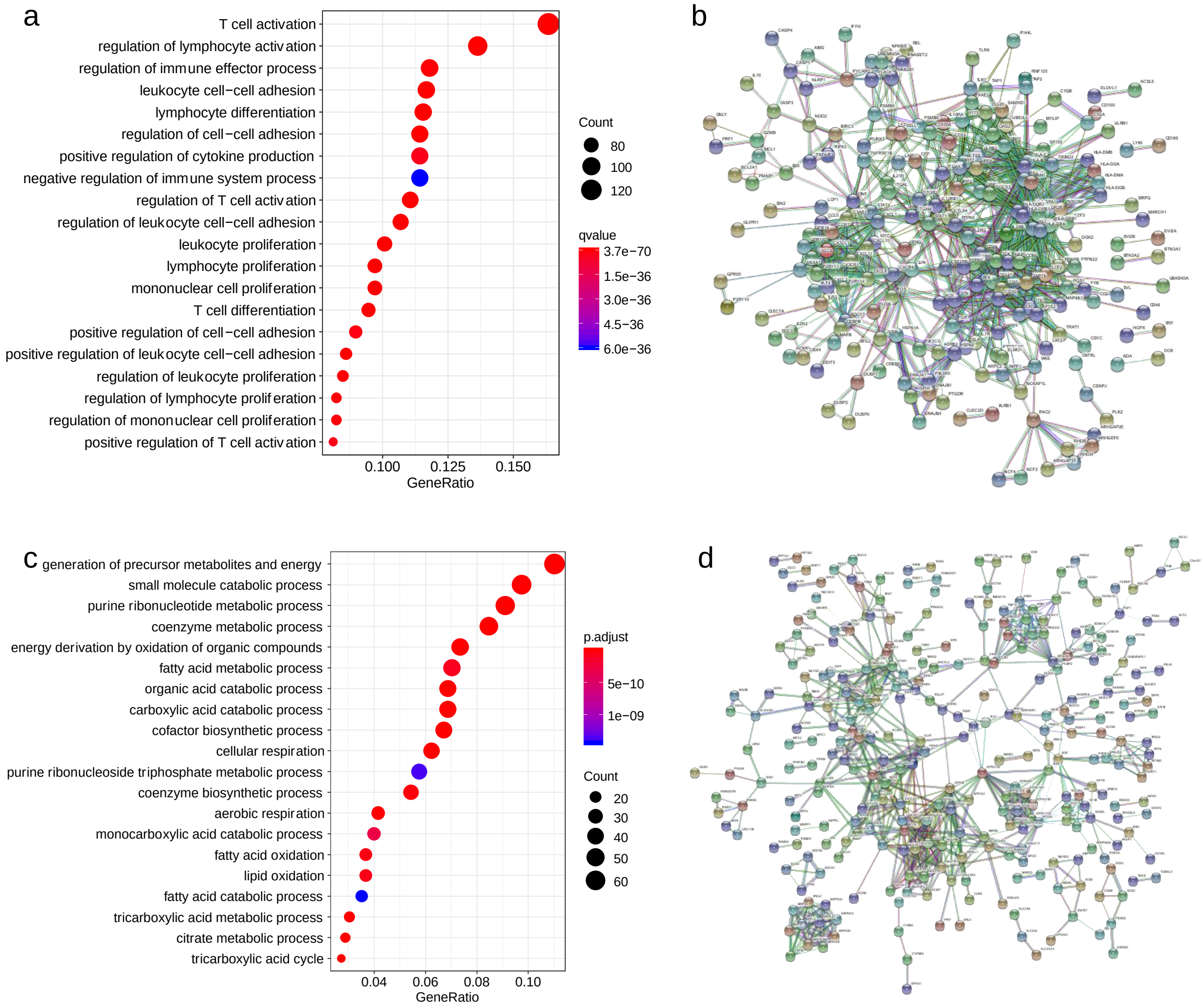

The top 20 GO terms enriched among (a) up- and (c) down-regulated genes in AR vs Normal analysis. Functional networking with STRING for the differentially expressed (b) up- and (d) down-regulated genes. We used the minimum required interaction score equal to 0.9. The edges mean a type of interaction evidence. The colors mean as follows. The known interactions are cyan for curated databases and magenta for experimentally determined interactions. The predicted interactions are represented in green for gene neighborhood, red for gene fusions, and blue for gene co-occurrence. The other types of interactions are in lime based on text mining, black on co-expression, and indigo on protein homology.

© 2021 Rychkov D et al. *JAMA* Network Open.

eFigure 5. Heatmap of Enrichment Scores of Significant Cell Types From the AR vs Normal Comparison

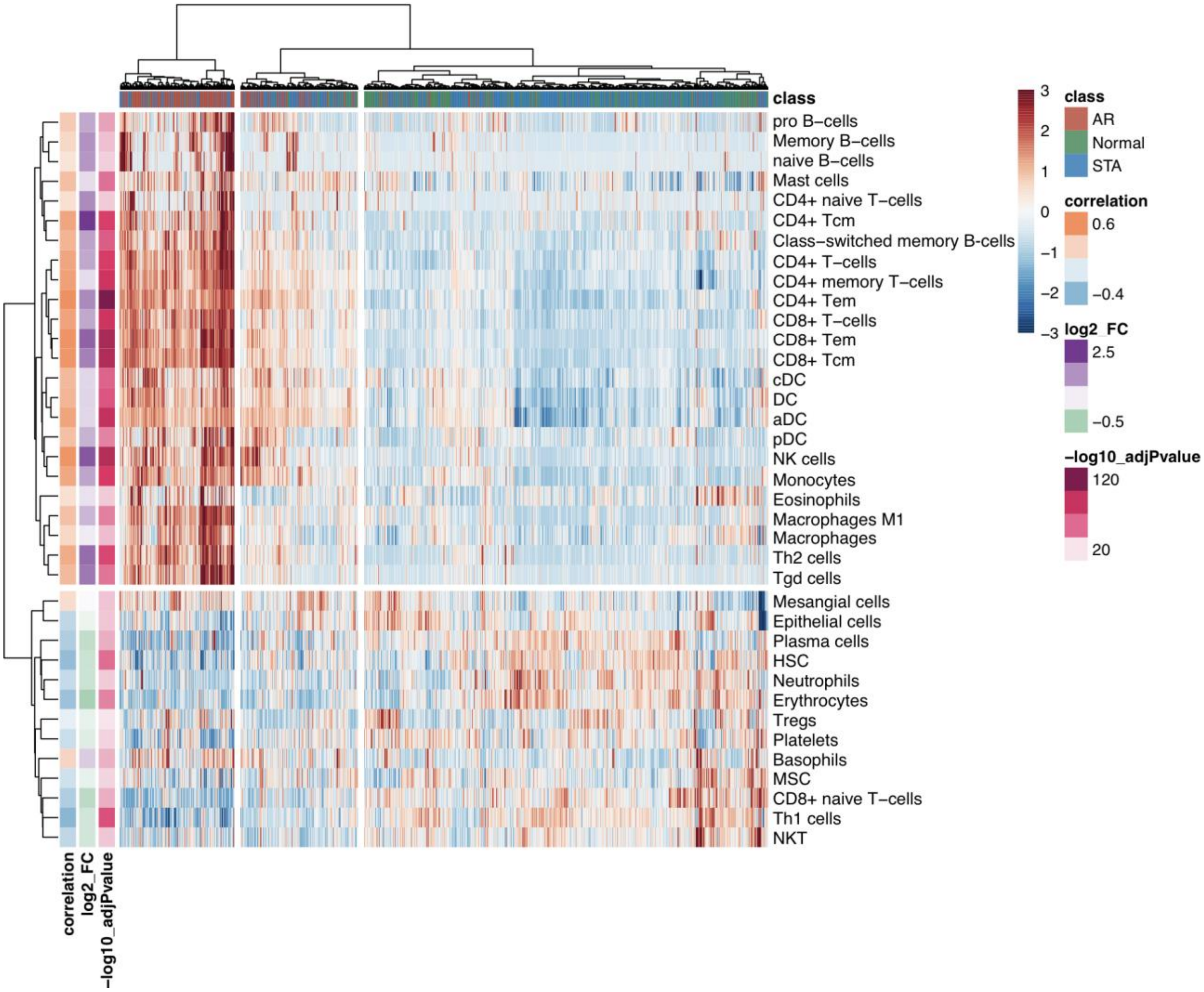

The colored vertical bars represent results from AR vs Normal analysis. The plot combines all AR, hSTA, and Normal samples and shows clustering of some hSTA samples together with AR hinting to possible hidden inflammation processes going in those grafts.

© 2021 Rychkov D et al. *JAMA* Network Open.

eFigure 6. Plots of Feature Selected Genes and Cell Types for all AR and Normal Samples

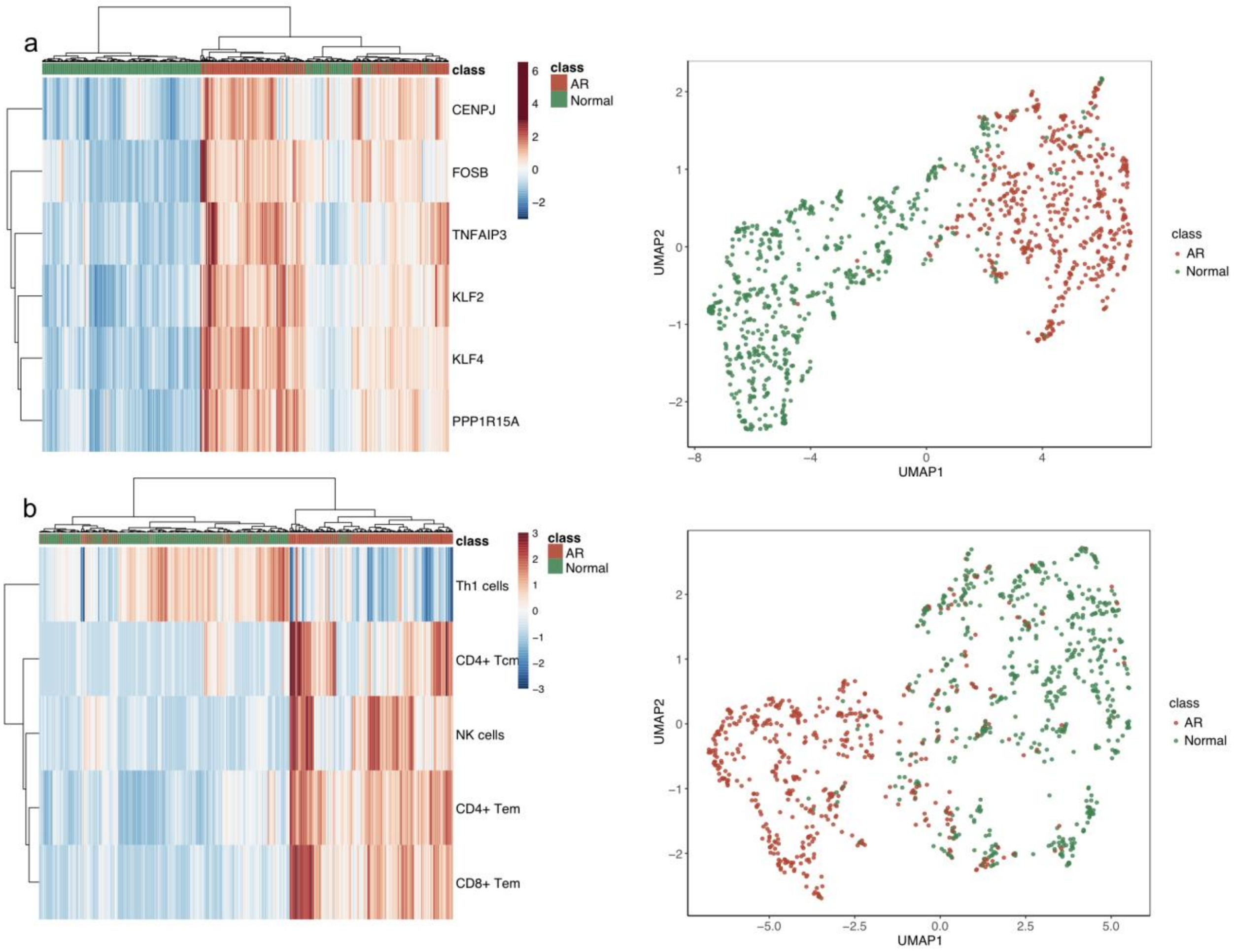

Heatmap and UMAP plot of (a) feature selected gene expression and (b) feature selected cell types.

© 2021 Rychkov D et al. *JAMA* Network Open.

eFigure 7. AUROC and AUCPR Plots of Feature Selected Genes, Cell Types and InstaScore

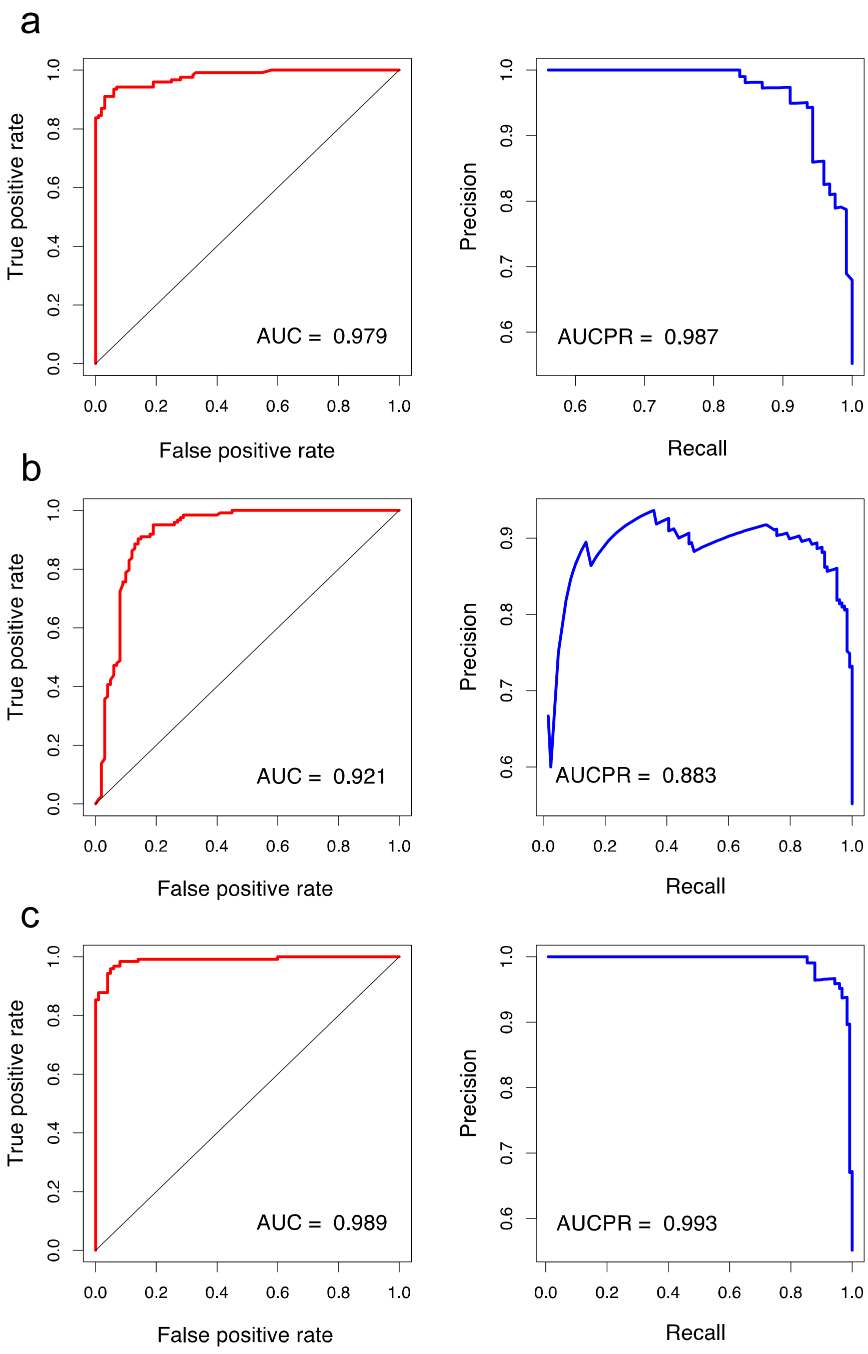

© 2021 Rychkov D et al. *JAMA* Network Open.

eFigure 8. Combined Benchmark Based on *P*-Value, Delta Statistic and the Percentage of Variability for Batch Correction Methods Tested

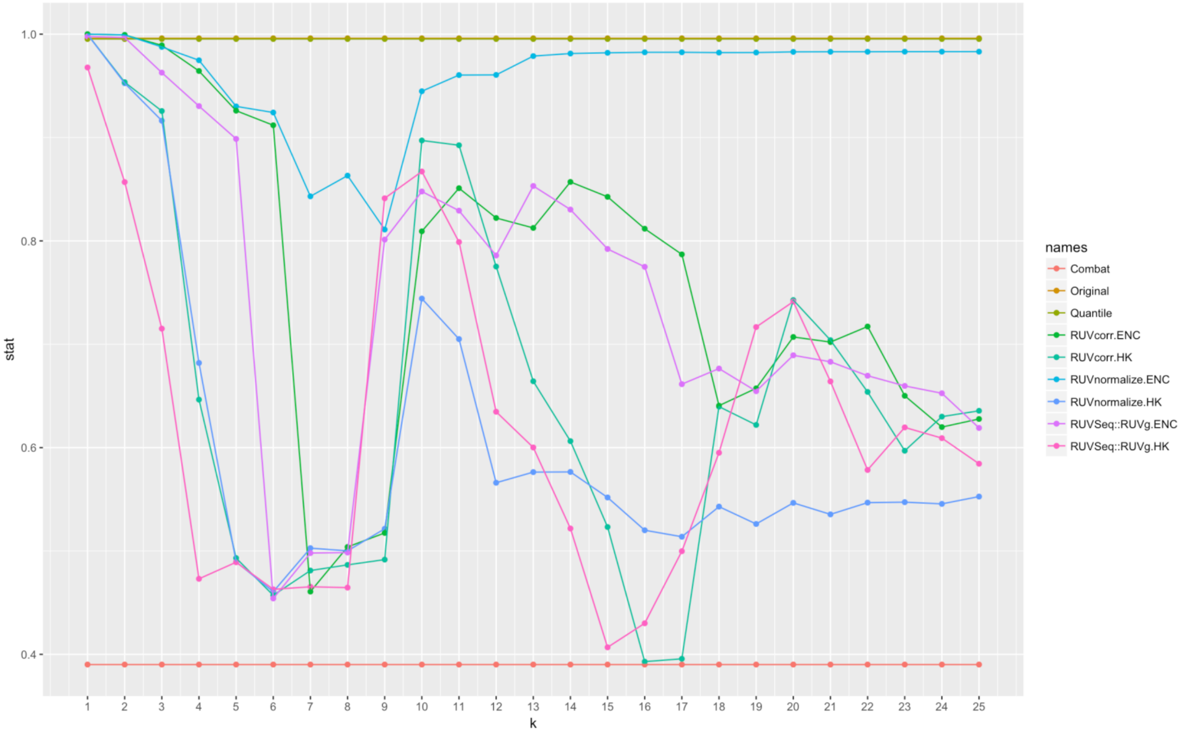

HK – Housekeeping genes, ENC – Empirical Negative Control genes

© 2021 Rychkov D et al. *JAMA* Network Open.

eTable 1. Datasets Collected From Gene Expression Omnibus (GEO)

Study	PMID	Platform	Probes	Total	AR	STA	Normal	Year	Country
GSE343	12853585	GPL271 LC-17 & GPL272 LC-20	28032 & 37632	41	26	0	15	2003	USA
GSE1563[a]	15307835	GPL8300 [HG_U95Av2] Affymetrix Human Genome U95 Version 2	12625	31	7	10	9	2004	USA
GSE1743[a]	15476476	GPL96 [HG-U133A] Affymetrix Human Genome U133A	22283	41	0	41	0	2004	USA
GSE7392	17397532	GPL570 [HG-U133_Plus_2] Affymetrix Human Genome U133 Plus 2.0	54675	22	0	7	15	2007	USA
GSE9493	19017305	GPL570 [HG-U133_Plus_2] Affymetrix Human Genome U133 Plus 2.0	54675	56	21	21	14	2007	Switzerland
GSE10419	20525436	GPL887 Agilent-012097 Human 1A Microarray (V2) G4110B	22153	29	0	29	0	2008	Japan
GSE11166	19443638	GPL570 [HG-U133_Plus_2] Affymetrix Human Genome U133 Plus 2.0	54675	49	1	27	21	2008	USA
GSE14328	20150539	GPL570 [HG-U133_Plus_2] Affymetrix Human Genome U133 Plus 2.0	54675	36	18	18	0	2009	USA
GSE14700[a]	20713790	GPL8150 Stanford Microarray Functional Genomics Homo sapiens 34.6K	34599	40	0	0	40	2009	Austria
GSE21374	20501945	GPL570 [HG-U133_Plus_2] Affymetrix Human Genome U133 Plus 2.0	54675	282	76	206	0	2010	Canada
GSE22459	20813870	GPL570 [HG-U133_Plus_2] Affymetrix Human Genome U133 Plus 2.0	54675	25	0	25	0	2010	USA
GSE25902	21881554	GPL570 [HG-U133_Plus_2] Affymetrix Human Genome U133 Plus 2.0	54675	88	27	37	24	2010	USA

© 2021 Rychkov D et al. *JAMA* Network Open.

GSE26578[a]	-	GPL9301 Zhejiang university human 449 oligonucleotide array	1347	85	34	51	0	2011	China
GSE30718	22343120	GPL570 [HG-U133_Plus_2] Affymetrix Human Genome U133 Plus 2.0	54675	19	0	11	8	2011	Canada
GSE34437	23437201	GPL570 [HG-U133_Plus_2] Affymetrix Human Genome U133 Plus 2.0	54675	36	17	7	12	2011	USA
GSE34748	22335458	GPL570 [HG-U133_Plus_2] Affymetrix Human Genome U133 Plus 2.0	54675	36	0	36	0	2011	USA
GSE36059	23356949	GPL570 [HG-U133_Plus_2] Affymetrix Human Genome U133 Plus 2.0	54675	410	122	280	8	2012	Canada
GSE43974	25427168	GPL10558 Illumina HumanHT-12 V4.0 expression beadchip	47323	463	0	112	351	2013	Netherlands
GSE44131	24030736	GPL6244 [HuGene-1_0-st] Affymetrix Human Gene 1.0 ST Array	29096	12	0	12	0	2013	USA
Study	PMID	Platform	Probes	Total	AR	STA	Normal	Year	Country
GSE47097	23763497	GPL6883 Illumina HumanRef-8 v3.0 expression beadchip	47323	40	36	4	0	2013	Netherlands
GSE48581	23915426	GPL570 [HG-U133_Plus_2] Affymetrix Human Genome U133 Plus 2.0	54675	306	78	222	6	2013	Canada
GSE50058	24127489	GPL570 [HG-U133_Plus_2] Affymetrix Human Genome U133 Plus 2.0	54675	56	31	25	0	2013	USA
GSE50084	26484130	GPL6244 [HuGene-1_0-st] Affymetrix Human Gene 1.0 ST Array	29096	48	28	20	0	2013	USA
GSE52694	26176825	GPL10558 Illumina HumanHT-12 V4.0 expression beadchip	23719	14	0	14	0	2013	Czech Republic
GSE53605	24698514	GPL571 [HG-U133A_2] Affymetrix Human Genome U133A 2.0	22277	31	13	18	0	2013	USA
GSE53769	20819195	GPL16686 [HuGene-2_0-st] Affymetrix Human Gene 2.0 ST Array	53617	28	0	10	18	2013	Austria
GSE54888	-	GPL6244 [HuGene-1_0-st] Affymetrix Human Gene 1.0 ST	33252	54	0	0	54	2014	Brazil

© 2021 Rychkov D et al. *JAMA* Network Open.

GSE57387	27452608	GPL5175 [HuEx-1_0-st] Affymetrix Human Exon 1.0 ST	17881	104	0	104	0	2014	USA
GSE60807	26908771	GPL6480 Agilent-014850 Whole Human Genome Microarray 4x44K G4112F	41093	37	0	0	37	2014	Austria
GSE65326	25307039	GPL10558 Illumina HumanHT-12 V4.0 expression beadchip	47323	6	0	6	0	2015	Australia
GSE69677	27369853	GPL14951 Illumina HumanHT-12 WG-DASL V4.0 R2 expression beadchip	29360	76	24	0	52	2015	Italy
GSE72925	27140517	GPL570 [HG-U133_Plus_2] Affymetrix Human Genome U133 Plus 2.0	54675	46	10	36	0	2015	USA
GSE76882	26990570	GPL13158 [HT_HG-U133_Plus_PM] Affymetrix HT HG-U133+ PM	17564	182	83	99	0	2016	USA
GSE82337[a]	27225518	GPL10558 Illumina HumanHT-12 V4.0 expression beadchip	47323	78	0	78	0	2016	USA
GSE83486[a]	28436117	GPL10558 Illumina HumanHT-12 V4.0 expression beadchip	47323	60	?	?	0	2016	Norway
GSE98320[a]	28614805	GPL15207 [PrimeView] Affymetrix Human Gene Expression Array	49395	1208	?	?	0	2017	Canada
GSE109346[a]	-	GPL10558 Illumina HumanHT-12 V4.0 expression beadchip	47323	26	0	26	0	2018	Czech Republic

[a] Datasets not included into this study

© 2021 Rychkov D et al. *JAMA* Network Open.

eTable 2a. Upregulated Differentially Expressed Genes From SAM Analysis of Comparison of Acute Rejection to Normal Kidney Tissues

gene	raw p.value	q.value	Bonferroni adj. p-value	fold change
KLF4	0	0	0	2.98997063
TNFAIP3	0	0	0	2.8158084
CENPJ	0	0	0	2.7977016
KLF2	0	0	0	2.7489507
PPP1R15A	0	0	0	2.73240129
CD69	0	0	0	2.72655781
IER5	0	0	0	2.64068239
IRF1	0	0	0	2.62101798
CXCL9	0	0	0	2.57674401
PMAIP1	0	0	0	2.49996879
CXCL10	0	0	0	2.49878491
GBP1	0	0	0	2.45983835
CXCL11	0	0	0	2.43386819
PRF1	0	0	0	2.43312954
EVI2A	0	0	0	2.42265645
RGS1	0	0	0	2.41594949
ZC3H12A	0	0	0	2.4151211
IL10RA	0	0	0	2.40736767
PLAC8	0	0	0	2.40119639
MAP3K8	0	0	0	2.40089927
CTSS	0	0	0	2.39761543
HCP5	0	0	0	2.38530964
PTPRC	0	0	0	2.38466318
KLRD1	0	0	0	2.38176354
TNF	0	0	0	2.37953081

© 2021 Rychkov D et al. *JAMA* Network Open.

TRIM22	0	0	0	2.37940218
IRF8	0	0	0	2.37358637
CD48	0	0	0	2.37304314
BTG2	0	0	0	2.37162986
CD3D	0	0	0	2.36786309
NKG7	0	0	0	2.36695982
CREB5	0	0	0	2.36661777
HLA-DQB2	0	0	0	2.36364457
TAP1	0	0	0	2.36149907
RUNX3	0	0	0	2.35113981
LST1	0	0	0	2.34840646
BIN2	0	0	0	2.34566138
HLA-DPA1	0	0	0	2.33798069
ITGAL	0	0	0	2.33385739
CSF2RB	0	0	0	2.32885078
P2RX7	0	0	0	2.32479792
IL7R	0	0	0	2.32355007
ARHGAP25	0	0	0	2.32177361
CCL5	0	0	0	2.32053852
MCL1	0	0	0	2.31631316
CST7	0	0	0	2.31616257
CD52	0	0	0	2.31387121
IKZF1	0	0	0	2.30961855
IDO1	0	0	0	2.30943484
AIM2	0	0	0	2.30837824
UBD	0	0	0	2.30495914
LCP2	0	0	0	2.30422715
KLRB1	0	0	0	2.30390509
EVI2B	0	0	0	2.29552135

© 2021 Rychkov D et al. *JAMA* Network Open.

PSMB9	0	0	0	2.29262263
CD86	0	0	0	2.29258553
BTN3A1	0	0	0	2.28967261
BTN3A2	0	0	0	2.28821269
CD247	0	0	0	2.28565063
CD8A	0	0	0	2.2850551
DEPP1	0	0	0	2.28276845
STK17B	0	0	0	2.28200493
HLA-DOB	0	0	0	2.28074243
ANXA1	0	0	0	2.27219254
APOBEC3G	0	0	0	2.27113781
HLA-DPB1	0	0	0	2.2665779
MYO1F	0	0	0	2.26465237
GZMK	0	0	0	2.26222309
GPR18	0	0	0	2.26205506
BCL11B	0	0	0	2.2616775
CXCR4	0	0	0	2.25937535
CD2	0	0	0	2.25318882
RGS10	0	0	0	2.25285452
P2RY13	0	0	0	2.2491914
GPR65	0	0	0	2.24744526
SP140	0	0	0	2.2454099
AOAH	0	0	0	2.24370008
ADRB2	0	0	0	2.24320158
LY9	0	0	0	2.24133679
FOSB	0	0	0	2.24000744
ISG20	0	0	0	2.23911862
MXD1	0	0	0	2.23635215
RIPOR2	0	0	0	2.23627031

© 2021 Rychkov D et al. *JAMA* Network Open.

ITK	0	0	0	2.23363861
KLF6	0	0	0	2.23235675
DNAJB1	0	0	0	2.23096799
LILRB1	0	0	0	2.23086453
CD96	0	0	0	2.23032482
HLA-E	0	0	0	2.22476943
STAT4	0	0	0	2.22381082
CLIC2	0	0	0	2.2235431
GZMB	0	0	0	2.22202816
MNDA	0	0	0	2.2167427
GPR171	0	0	0	2.21539441
SLAMF8	0	0	0	2.2142648
IL2RB	0	0	0	2.20896132
MICB	0	0	0	2.20499951
WEE1	0	0	0	2.20455374
MARCH1	0	0	0	2.20291497
LYZ	0	0	0	2.18920863
CLEC10A	0	0	0	2.1802396
CXCL2	0	0	0	2.17895268
P2RY10	0	0	0	2.1683341
CASP1	0	0	0	2.16819752
GIMAP6	0	0	0	2.16746414
HLA-DMA	0	0	0	2.16708189
DUSP5	0	0	0	2.16613359
ST8SIA4	0	0	0	2.15984574
PTPN22	0	0	0	2.15949268
BIRC3	0	0	0	2.15651003
GPR183	0	0	0	2.15341854
LCK	0	0	0	2.15216208

© 2021 Rychkov D et al. *JAMA* Network Open.

HLA-DMB	0	0	0	2.15053615
IKZF3	0	0	0	2.14858732
CBX4	0	0	0	2.14598088
SP110	0	0	0	2.14380387
ACSL5	0	0	0	2.14223594
APOL3	0	0	0	2.13987992
SELL	0	0	0	2.13968371
CELF2	0	0	0	2.13866275
SP140L	0	0	0	2.13763051
CSTA	0	0	0	2.13088689
GNLY	0	0	0	2.13065208
JUNB	0	0	0	2.13053963
RIPK2	0	0	0	2.1286435
PSMB8	0	0	0	2.12774922
JUN	0	0	0	2.12526537
TNFAIP8	0	0	0	2.12453999
RAC2	0	0	0	2.11800249
MAP4K1	0	0	0	2.11599607
NFKBIE	0	0	0	2.1131527
NOD2	0	0	0	2.1119409
CLEC4A	0	0	0	2.11130621
TYROBP	0	0	0	2.11073863
PLK2	0	0	0	2.10985017
CD1C	0	0	0	2.10894258
ARHGAP15	0	0	0	2.10693429
CORO1A	0	0	0	2.10679548
MS4A6A	0	0	0	2.10658045
SLA	0	0	0	2.10636427
NCF2	0	0	0	2.10529116

© 2021 Rychkov D et al. *JAMA* Network Open.

FOXJ1	0	0	0	2.10425914
IFI16	0	0	0	2.10310793
HSPA1A	0	0	0	2.10295144
PELI1	0	0	0	2.10186002
TLR2	0	0	0	2.10115727
SH2D1A	0	0	0	2.09767889
MYC	0	0	0	2.09732699
FCN1	0	0	0	2.09723406
LAX1	0	0	0	2.09504128
PTPRCAP	0	0	0	2.09413584
RHOB	0	0	0	2.09263867
SIRPG	0	0	0	2.0911939
IFNG	0	0	0	2.08976079
HCK	0	0	0	2.08955668
MAFB	0	0	0	2.08872583
CLEC2D	0	0	0	2.08446512
TRAF3IP3	0	0	0	2.08387276
ZFP36L2	0	0	0	2.08310434
PRKCB	0	0	0	2.07722812
SASH3	0	0	0	2.07441304
CD38	0	0	0	2.07378644
CD160	0	0	0	2.07176452
DPEP2	0	0	0	2.07159006
ADGRE5	0	0	0	2.07126431
PYCARD	0	0	0	2.07070006
ADCY7	0	0	0	2.06925373
HLA-DRA	0	0	0	2.06894425
UBE2L6	0	0	0	2.06593758
CRLF3	0	0	0	2.06126017

© 2021 Rychkov D et al. *JAMA* Network Open.

DOK2	0	0	0	2.06030902
VMP1	0	0	0	2.06020704
TENT5A	0	0	0	2.05872026
LYN	0	0	0	2.05847505
EZH2	0	0	0	2.05703329
CCNL1	0	0	0	2.05521972
ITGAX	0	0	0	2.05505202
INPP5D	0	0	0	2.05274694
RNF125	0	0	0	2.05195938
DYRK2	0	0	0	2.04307727
FAM53C	0	0	0	2.04258896
IRF7	0	0	0	2.04170872
HLA-B	0	0	0	2.04143156
LAT2	0	0	0	2.04058043
APOL1	0	0	0	2.0403936
LAPTM5	0	0	0	2.04032522
FYB1	0	0	0	2.0400202
CLEC7A	0	0	0	2.03907773
TRAT1	0	0	0	2.03828489
IL16	0	0	0	2.0380657
RRAD	0	0	0	2.03760206
FAM49A	0	0	0	2.03460842
RUBCNL	0	0	0	2.03453033
SAMSN1	0	0	0	2.03442298
PLEKHO2	0	0	0	2.03227139
RTN1	0	0	0	2.02967175
MCUB	0	0	0	2.0294391
CD300A	0	0	0	2.02925183
UBASH3A	0	0	0	2.02613114

© 2021 Rychkov D et al. *JAMA* Network Open.

PIK3CG	0	0	0	2.02611959
KIAA1551	0	0	0	2.0260504
PRDM1	0	0	0	2.02375524
RASSF2	0	0	0	2.02326564
TCIRG1	0	0	0	2.02210548
ADAMDEC1	0	0	0	2.01991221
MYLIP	0	0	0	2.0186271
ITGAM	0	0	0	2.01630828
PVRIG	0	0	0	2.01473335
STAT1	0	0	0	2.01429309
TLR8	0	0	0	2.01404994
PTGDR	0	0	0	2.01108627
CFP	0	0	0	2.01085542
BTN2A2	0	0	0	2.01038137
SOCS3	0	0	0	2.00865113
C1QA	0	0	0	2.00796476
SAMHD1	0	0	0	2.00746237
FGR	0	0	0	2.0054924
CASP3	0	0	0	2.00509585
IL21R	0	0	0	2.0043075
ITGB2	0	0	0	2.00417258
POU2AF1	0	0	0	2.00377665
PTPN6	0	0	0	2.0020436
CD27	0	0	0	2.00177412
RGS19	0	0	0	1.99981528
GBP2	0	0	0	1.99785514
ELF4	0	0	0	1.99639093
DDIT3	0	0	0	1.99400543
LILRB2	0	0	0	1.99287834

© 2021 Rychkov D et al. *JAMA* Network Open.

GIMAP4	0	0	0	1.9920831
ZAP70	0	0	0	1.99182192
HLA-DOA	0	0	0	1.99131317
CD8B	0	0	0	1.98886458
IGSF6	0	0	0	1.98812839
DNAJA1	0	0	0	1.98733308
GCNT1	0	0	0	1.98698841
FCMR	0	0	0	1.98692169
DNAJB4	0	0	0	1.98534535
OAS2	0	0	0	1.98424631
PTPRE	0	0	0	1.98342392
ST3GAL5	0	0	0	1.98159439
P2RY14	0	0	0	1.97909875
REL	0	0	0	1.97905329
SRF	0	0	0	1.97313036
CX3CR1	0	0	0	1.97167246
LAIR1	0	0	0	1.97127262
IL2RG	0	0	0	1.97107721
CD180	0	0	0	1.9709026
ELOVL1	0	0	0	1.96958038
HSPA8	0	0	0	1.96898397
CXCL1	0	0	0	1.96815369
DUSP1	0	0	0	1.96663177
TRIB1	0	0	0	1.96518202
NMI	0	0	0	1.96096383
KLRC3	0	0	0	1.96068833
FGL2	0	0	0	1.96065735
HBEGF	0	0	0	1.96027009
HLA-DRB1	0	0	0	1.9599303

© 2021 Rychkov D et al. *JAMA* Network Open.

DOCK10	0	0	0	1.9593274
TLR7	0	0	0	1.95783505
LY86	0	0	0	1.95747111
LCP1	0	0	0	1.95485984
BCL2A1	0	0	0	1.95022069
DUSP6	0	0	0	1.9482675
NCF4	0	0	0	1.94488048
ALOX5	0	0	0	1.94476151
CX3CL1	0	0	0	1.94438257
WIPF1	0	0	0	1.94412603
C3AR1	0	0	0	1.94320305
CD3G	0	0	0	1.94275676
ARPC2	0	0	0	1.94275454
APOBEC3A	0	0	0	1.94216227
ADA	0	0	0	1.94184678
DUSP2	0	0	0	1.94061147
XAF1	0	0	0	1.94040151
CD37	0	0	0	1.93799495
CTSW	0	0	0	1.93610919
NLRP1	0	0	0	1.93558212
ZFP36L1	0	0	0	1.93447777
PSTPIP2	0	0	0	1.93439906
HLA-C	0	0	0	1.93078022
IL12RB1	0	0	0	1.92876709
SRGN	0	0	0	1.92844358
MAN2B1	0	0	0	1.92774418
TNFRSF1B	0	0	0	1.92743362
TMSB10	0	0	0	1.92659658
CD84	0	0	0	1.92429219

© 2021 Rychkov D et al. *JAMA* Network Open.

PLEKHO1	0	0	0	1.92315891
LAMP3	0	0	0	1.92306466
LAT	0	0	0	1.92271261
CCR7	0	0	0	1.92056913
CNTRL	0	0	0	1.91987418
TAP2	0	0	0	1.91852928
VAMP1	0	0	0	1.91840413
CAMK1D	0	0	0	1.91706925
THEMIS2	0	0	0	1.91592596
CXCL8	0	0	0	1.91498407
PARP12	0	0	0	1.91288996
PIK3R5	0	0	0	1.91143136
CASP4	0	0	0	1.91028827
MCTP1	0	0	0	1.9091806
CEBPB	0	0	0	1.90915733
ARL4C	0	0	0	1.90817935
ACKR1	0	0	0	1.9068612
PSTPIP1	0	0	0	1.90549377
EVL	0	0	0	1.90530416
SP100	0	0	0	1.90249529
ERN1	0	0	0	1.90238376
RTP4	0	0	0	1.90175352
IFIT3	0	0	0	1.90155478
HSPH1	0	0	0	1.90127208
C1QB	0	0	0	1.89829302
NCKAP1L	0	0	0	1.8982184
AHR	0	0	0	1.89633779
SLC20A1	0	0	0	1.89576799
DHRS9	0	0	0	1.89386659

© 2021 Rychkov D et al. *JAMA* Network Open.

CHIC2	0	0	0	1.89354804
MS4A4A	0	0	0	1.89331474
SLFN12	0	0	0	1.89263102
ADGRE2	0	0	0	1.89047958
RNASET2	0	0	0	1.88729402
FCGR2B	0	0	0	1.88665576
HOPX	0	0	0	1.88659649
IFI44L	0	0	0	1.88521157
AP1S2	0	0	0	1.88454226
WAS	0	0	0	1.88348658
AIF1	0	0	0	1.88286408
GLIPR1	0	0	0	1.88249235
SERTAD2	0	0	0	1.8823785
DAPP1	0	0	0	1.88159526
FAM129A	0	0	0	1.88137884
SDC3	0	0	0	1.87967227
SIGLEC1	0	0	0	1.87925645
DCK	0	0	0	1.87737708
RGS16	0	0	0	1.87641639
ELMO1	0	0	0	1.87600998
BAZ1A	0	0	0	1.87593474
IGLL1	0	0	0	1.87570879
TARP	0	0	0	1.87544739
CTLA4	0	0	0	1.87449183
BID	0	0	0	1.87368882
ARHGEF6	0	0	0	1.87049913
BTK	0	0	0	1.86868858
RND3	0	0	0	1.86855943
WARS	0	0	0	1.86585704

© 2021 Rychkov D et al. *JAMA* Network Open.

RHOH	0	0	0	1.86556438
FOS	0	0	0	1.86544956
IL18RAP	0	0	0	1.86529522
MX2	0	0	0	1.8647553
CD1D	0	0	0	1.86404656
CXCR6	0	0	0	1.86353797
KLRF1	0	0	0	1.8628973
OGFRL1	0	0	0	1.86063546
PLA2G7	0	0	0	1.86001606
H2AFY	0	0	0	1.85773294
PSMB10	0	0	0	1.85673482
SRSF7	0	0	0	1.8554265
ARHGDIB	0	0	0	1.85440944
DUSP4	0	0	0	1.85437137
CD40	0	0	0	1.85384103
CD33	0	0	0	1.85306627
IFIT2	0	0	0	1.85287826
SERPINH1	0	0	0	1.85195787
DDB2	0	0	0	1.85154538
TRAF5	0	0	0	1.85064829
IFFO1	0	0	0	1.84865504
PRKCH	0	0	0	1.84639427
BTG1	0	0	0	1.84552933
PTPN7	0	0	0	1.84410823
TLR4	0	0	0	1.84382548
KIF21B	0	0	0	1.84370578
CXCL13	0	0	0	1.84273866
JCHAIN	0	0	0	1.84083479
SLAMF7	0	0	0	1.84047916

© 2021 Rychkov D et al. *JAMA* Network Open.

CLK1	0	0	0	1.83892865
TMEM131L	0	0	0	1.83535327
MARCKSL1	0	0	0	1.83431175
VAV1	0	0	0	1.83420174
SECTM1	0	0	0	1.83406348
CD44	0	0	0	1.83359946
FN1	0	0	0	1.83323341
DUSP10	0	0	0	1.83313566
HLA-DQA1	0	0	0	1.83310857
MARCKS	0	0	0	1.83110396
ZFP36	0	0	0	1.83106185
DGKA	0	0	0	1.83052584
SLAMF1	0	0	0	1.8294436
OASL	0	0	0	1.82666909
HMGB2	0	0	0	1.82484549
BASP1	0	0	0	1.82397046
CTSC	0	0	0	1.8233423
EFHD2	0	0	0	1.82321991
ZC3HAV1	0	0	0	1.82068014
PIK3CD	0	0	0	1.82056002
SIT1	0	0	0	1.81895582
CSK	0	0	0	1.81866637
LTB	0	0	0	1.81845229
APOC1	0	0	0	1.81671309
TCF7	0	0	0	1.81630922
CXorf21	0	0	0	1.81411418
BATF	0	0	0	1.81388217
RASSF1	0	0	0	1.81345543
TSC22D1	0	0	0	1.81320778

© 2021 Rychkov D et al. *JAMA* Network Open.

VNN2	0	0	0	1.81227913
LAG3	0	0	0	1.8122329
PLA1A	0	0	0	1.81015378
TNFAIP2	0	0	0	1.80815742
TNFRSF17	0	0	0	1.80546766
VIM	0	0	0	1.80541718
IFI44	0	0	0	1.80271739
FCHSD2	0	0	0	1.80231917
TNFRSF9	0	0	0	1.80129713
GIT2	0	0	0	1.80113696
RAB27A	0	0	0	1.79455542
IFITM1	0	0	0	1.79287394
CPA3	0	0	0	1.79108156
FCER1G	0	0	0	1.78901746
SOCS1	0	0	0	1.78458929
CSF3R	0	0	0	1.78449721
MZB1	0	0	0	1.78433334
MKNK2	0	0	0	1.78210981
TLR1	0	0	0	1.7820085
B2M	0	0	0	1.78142226
EBI3	0	0	0	1.78005656
GMIP	0	0	0	1.78000393
PTAFR	0	0	0	1.77706039
ATP8B2	0	0	0	1.77703527
CBFA2T3	0	0	0	1.77658697
FASLG	0	0	0	1.77630409
CD74	0	0	0	1.77562647
IFNAR2	0	0	0	1.7732499
CCL19	0	0	0	1.77252545

© 2021 Rychkov D et al. *JAMA* Network Open.

NELL2	0	0	0	1.77202311
FXYD5	0	0	0	1.77185315
BRD2	0	0	0	1.76772958
JAK3	0	0	0	1.76724512
LAIR2	0	0	0	1.76502577
DDX60	0	0	0	1.76476678
CD7	0	0	0	1.76360243
NDC80	0	0	0	1.76311006
PRPF38B	0	0	0	1.76224003
FAR2	0	0	0	1.75902018
ARPC1B	0	0	0	1.75835304
MAP3K3	0	0	0	1.75834941
CKLF	0	0	0	1.75797445
MS4A1	0	0	0	1.75783386
ARNTL2	0	0	0	1.75621308
TNFSF8	0	0	0	1.75446324
RRM2	0	0	0	1.75373194
EGR1	0	0	0	1.75190521
GRK3	0	0	0	1.75167088
SIDT1	0	0	0	1.75084914
APBB1IP	0	0	0	1.74973631
APOBEC3C	0	0	0	1.74959712
CD40LG	0	0	0	1.74823959
BTN2A1	0	0	0	1.74794306
MICALL2	0	0	0	1.74741849
PSME1	0	0	0	1.74648474
TES	0	0	0	1.74638962
ZBP1	0	0	0	1.74588639
SDCBP	0	0	0	1.7454835

© 2021 Rychkov D et al. *JAMA* Network Open.

CD72	0	0	0	1.7454823
TGFBI	0	0	0	1.74422384
MTMR14	0	0	0	1.74329528
CARD8	0	0	0	1.74304429
DENND1C	0	0	0	1.74239717
TSPAN32	0	0	0	1.73972072
RASA2	0	0	0	1.73971197
ST8SIA1	0	0	0	1.73814596
CCL8	0	0	0	1.73702165
SERPINB1	0	0	0	1.73620964
DUSP8	0	0	0	1.73578473
IRF4	0	0	0	1.73526639
FPR3	0	0	0	1.73512889
TCIM	0	0	0	1.73505704
BLM	0	0	0	1.73263421
CD79B	0	0	0	1.73097172
GNA13	0	0	0	1.72706217
BCL11A	0	0	0	1.72641206
WFDC2	0	0	0	1.72593419
ELF1	0	0	0	1.72581194
C2CD2	0	0	0	1.72404254
RAB31	0	0	0	1.72298895
LIMD2	0	0	0	1.72189114
CCNA2	0	0	0	1.71960077
ELF3	0	0	0	1.7195446
FOLR2	0	0	0	1.71948655
CYTH4	0	0	0	1.71866983
MYB	0	0	0	1.71823476
NXF1	0	0	0	1.71791097

© 2021 Rychkov D et al. *JAMA* Network Open.

LDLR	0	0	0	1.71763236
SEPT6	0	0	0	1.71577512
TTC13	0	0	0	1.71560699
CHST11	0	0	0	1.71542874
CAV1	0	0	0	1.71540046
CCDC69	0	0	0	1.71531691
DNMT1	0	0	0	1.71501879
JAK2	0	0	0	1.71054734
LPAR6	0	0	0	1.71008926
MYD88	0	0	0	1.70690211
VAMP5	0	0	0	1.70675365
FSCN1	0	0	0	1.70633955
MIS12	0	0	0	1.70426291
TRAFD1	0	0	0	1.70177249
BMP2	0	0	0	1.7014756
STX11	0	0	0	1.70103888
EED	0	0	0	1.70083381
RASGRP1	0	0	0	1.70070945
LUM	0	0	0	1.70058634
SERPING1	0	0	0	1.70050886
RFX5	0	0	0	1.69983355
RBM15	0	0	0	1.69935909
PILRA	0	0	0	1.69865201
VPS13C	0	0	0	1.69859385
LPAR1	0	0	0	1.69726336
IRF9	0	0	0	1.69660496
NCAPG	0	0	0	1.69583141
TRAF1	0	0	0	1.69482668
CH25H	0	0	0	1.69392185

© 2021 Rychkov D et al. *JAMA* Network Open.

SPN	0	0	0	1.69334013
HERC5	0	0	0	1.69202797
LOXL1	0	0	0	1.69136803
MSX1	0	0	0	1.69052508
TNFRSF25	0	0	0	1.6902624
CD3E	0	0	0	1.69017111
CYLD	0	0	0	1.68968309
RAP2B	0	0	0	1.68963492
TUT7	0	0	0	1.68912208
MTHFD2	0	0	0	1.6880102
CD80	0	0	0	1.68663534
VSIG4	0	0	0	1.6863206
S100A6	0	0	0	1.68574209
UBA7	0	0	0	1.68222646
ANXA2	0	0	0	1.68172598
CSF1R	0	0	0	1.68013518
CAMK4	0	0	0	1.67681373
KCNJ2	0	0	0	1.67648154
N4BP2L1	0	0	0	1.67633222
S100PBP	0	0	0	1.67347362
REEP4	0	0	0	1.67344001
APOL6	0	0	0	1.67022575
DLGAP5	0	0	0	1.66820897
RSRP1	0	0	0	1.66807055
SOX4	0	0	0	1.66792417
ATAD2	0	0	0	1.66751303
CENPU	0	0	0	1.66747276
CD19	0	0	0	1.66687771
IRAK3	0	0	0	1.66672951

© 2021 Rychkov D et al. *JAMA* Network Open.

CCND3	0	0	0	1.6665844
FILIP1L	0	0	0	1.66653097
UCP2	0	0	0	1.66458912
PLXNC1	0	0	0	1.66453026
NUSAP1	0	0	0	1.66404752
STK26	0	0	0	1.66303644
FLI1	0	0	0	1.6619662
ERAP2	0	0	0	1.66131371
CXCR3	0	0	0	1.66070592
RSAD2	0	0	0	1.65988436
PABPC4	0	0	0	1.65970338
LMNB1	0	0	0	1.65930683
C1R	0	0	0	1.65830752
HELLS	0	0	0	1.65749816
FKBP11	0	0	0	1.65567463
CD4	0	0	0	1.6553146
ARAP2	0	0	0	1.65520485
CCL18	0	0	0	1.6550502
PCLAF	0	0	0	1.65472466
ITM2A	0	0	0	1.65344785
ZNF266	0	0	0	1.65257028
MCM7	0	0	0	1.65173926
CLIC3	0	0	0	1.65166733
ARHGAP22	0	0	0	1.64985989
TENT5C	0	0	0	1.64978873
KLRG1	0	0	0	1.64773556
HECA	0	0	0	1.64736233
DOK3	0	0	0	1.64595579
GNPTAB	0	0	0	1.64497669

© 2021 Rychkov D et al. *JAMA* Network Open.

ASAP1	0	0	0	1.64409113
CD47	0	0	0	1.6426827
HIST1H3D	0	0	0	1.64205208
PTGER4	0	0	0	1.64113961
SLC15A3	0	0	0	1.64061527
TRA2A	0	0	0	1.64005512
DTL	0	0	0	1.63962487
DCLRE1C	0	0	0	1.63884978
ZNF165	0	0	0	1.63803652
ACAP1	0	0	0	1.63735384
RASGRP2	0	0	0	1.63733558
KIF20A	0	0	0	1.63641669
TOP2A	0	0	0	1.63571679
ELK3	0	0	0	1.63562165
VPS37B	0	0	0	1.63549297
GSDMB	0	0	0	1.63544187
TCF4	0	0	0	1.63518521
MFNG	0	0	0	1.63486088
CBLB	0	0	0	1.63474222
TRIM56	0	0	0	1.63348557
VCAN	0	0	0	1.6302336
LTF	0	0	0	1.62950828
MDK	0	0	0	1.629157
IFNGR2	0	0	0	1.62915645
CEBPA	0	0	0	1.62831425
IFI27	0	0	0	1.62819676
BUB1	0	0	0	1.6280698
TNC	0	0	0	1.62644798
NNMT	0	0	0	1.62527629

© 2021 Rychkov D et al. *JAMA* Network Open.

CD244	0	0	0	1.62509357
CEP55	0	0	0	1.62488989
COL3A1	0	0	0	1.62386121
ADAP2	0	0	0	1.62285782
RHOG	0	0	0	1.62264651
PLXND1	0	0	0	1.62261016
IRF2	0	0	0	1.62224868
ISG15	0	0	0	1.62224174
IMPDH1	0	0	0	1.62109375
SNW1	0	0	0	1.62073662
MMP7	0	0	0	1.620502
CARD9	0	0	0	1.61909898
GNAI2	0	0	0	1.61904287
IL12B	0	0	0	1.61862507
CD79A	0	0	0	1.61840524
CHFR	0	0	0	1.61663011
AKAP13	0	0	0	1.61471276
OAS3	0	0	0	1.61390275
MEOX1	0	0	0	1.61332761
TDO2	0	0	0	1.61326664
OLFML2B	0	0	0	1.61166499
UBE2C	0	0	0	1.61067853
KPNB1	0	0	0	1.61053089
CP	0	0	0	1.61036798
PDCD4	0	0	0	1.60856048
TYMP	0	0	0	1.60849228
CBR3	0	0	0	1.60826161
TSC22D2	0	0	0	1.60811616
FAM111A	0	0	0	1.6066557

© 2021 Rychkov D et al. *JAMA* Network Open.

NECAP2	0	0	0	1.60571054
PIK3IP1	0	0	0	1.60430458
GFOD1	0	0	0	1.6036544
GZMM	0	0	0	1.60351277
CYTH1	0	0	0	1.60332448
ATM	0	0	0	1.6030466
INPP4B	0	0	0	1.60263985
DENND2D	0	0	0	1.60204307
STK4	0	0	0	1.60157483
LAP3	0	0	0	1.60133438
TNFRSF4	0	0	0	1.60087427
EMP3	0	0	0	1.60021588
ADRA2A	0	0	0	1.60007262
PIM1	0	0	0	1.59987341
C21orf91	0	0	0	1.59971125
LY96	0	0	0	1.59950292
MSL3	0	0	0	1.59927518
PTGER2	0	0	0	1.59901072
RPS11	0	0	0	1.59898459
CLEC4E	0	0	0	1.5985495
TPX2	0	0	0	1.59793469
C1S	0	0	0	1.59736586
FES	0	0	0	1.59660446
XBP1	0	0	0	1.59485273
MCAM	0	0	0	1.5946111
LBH	0	0	0	1.59378322
CENPA	0	0	0	1.59349213
BCL10	0	0	0	1.59321777
SH3BGRL3	0	0	0	1.59272262

© 2021 Rychkov D et al. *JAMA* Network Open.

NDEL1	0	0	0	1.59217166
IER2	0	0	0	1.59190133
CD1E	0	0	0	1.5916335
TRIM21	0	0	0	1.59113097
CMKLR1	0	0	0	1.59051555
GNA15	0	0	0	1.59005716
ERAP1	0	0	0	1.58978212
SAMD9	0	0	0	1.58959091
ETS1	0	0	0	1.58930415
GPSM3	0	0	0	1.58870132
ARPC5	0	0	0	1.58710684
PLSCR1	0	0	0	1.58670584
KIF14	0	0	0	1.58649839
PMP22	0	0	0	1.58647722
ZBED2	0	0	0	1.58630476
TUBA1A	0	0	0	1.58581074
PRR5L	0	0	0	1.58571366
CD226	0	0	0	1.5855409
TMEM156	0	0	0	1.58506789
PMCH	0	0	0	1.58481651
RNF19A	0	0	0	1.58461757
CCNE2	0	0	0	1.5844883
RBMS1	0	0	0	1.58444775
TAPBPL	0	0	0	1.58417677
PDLIM1	0	0	0	1.58382954
CDKN1B	0	0	0	1.58365374
BCL6	0	0	0	1.5832491
CD6	0	0	0	1.58174635
LFNG	0	0	0	1.58157241

© 2021 Rychkov D et al. *JAMA* Network Open.

NAGK	0	0	0	1.58039514
RIPK4	0	0	0	1.57852986
NABP1	0	0	0	1.57736644
HIST1H2BG	0	0	0	1.57670043
NTM	0	0	0	1.5763966
SAT1	0	0	0	1.57598707
TIPARP	0	0	0	1.57538386
FNTA	0	0	0	1.57536563
HSP90AA1	0	0	0	1.57456349
NR3C1	0	0	0	1.57382185
ZNF217	0	0	0	1.57327555
CCND2	0	0	0	1.57271228
TSC22D3	0	0	0	1.57224168
TNIP3	0	0	0	1.57191221
KIF4A	0	0	0	1.5712038
TAPBP	0	0	0	1.5691961
PRC1	0	0	0	1.56853573
PRKD2	0	0	0	1.56819464
CDKN2AIP	0	0	0	1.56647593
BACH2	0	0	0	1.56642363
BATF3	0	0	0	1.56609817
PABPC3	0	0	0	1.56603305
H2AFX	0	0	0	1.56234496
SELE	0	0	0	1.56216495
UBE2E1	0	0	0	1.56174541
CST3	0	0	0	1.56169459
HHEX	0	0	0	1.56125914
FCRL2	0	0	0	1.56038949
TIMP2	0	0	0	1.55960415

© 2021 Rychkov D et al. *JAMA* Network Open.

HDC	0	0	0	1.55941959
SLC16A6	0	0	0	1.55881788
C3	0	0	0	1.55828046
KIF11	0	0	0	1.55739121
IL7	0	0	0	1.55707654
KLF11	0	0	0	1.55682229
NINJ2	0	0	0	1.55679518
NPDC1	0	0	0	1.55666889
TMC6	0	0	0	1.55608342
MMP9	0	0	0	1.55601107
IL1B	0	0	0	1.55480441
IL27RA	0	0	0	1.5547195
SSR2	0	0	0	1.55462966
PNOC	0	0	0	1.55384537
GAS7	0	0	0	1.55366868
MYO9B	0	0	0	1.55296802
CCL11	0	0	0	1.55293404
AGBL2	0	0	0	1.55212726
GEM	0	0	0	1.55160141
GTF2B	0	0	0	1.55096697
SETX	0	0	0	1.55092221
NFKB1	0	0	0	1.55058188
PBK	0	0	0	1.54964689
SELP	0	0	0	1.54915175
ZEB2	0	0	0	1.54855891
ORC6	0	0	0	1.54799812
TIMP1	0	0	0	1.54778926
BLK	0	0	0	1.54745075
GLA	0	0	0	1.54694853

© 2021 Rychkov D et al. *JAMA* Network Open.

DUSP22	0	0	0	1.54549389
KBTBD2	0	0	0	1.54525509
IL2RA	0	0	0	1.54518642
FBXO5	0	0	0	1.5451359
KIF15	0	0	0	1.54475812
MGAT1	0	0	0	1.54469839
TOB2	0	0	0	1.54406523
ASPM	0	0	0	1.54363838
ADAMTS1	0	0	0	1.5431586
CITED2	0	0	0	1.54298474
CENPM	0	0	0	1.54256655
CDH5	0	0	0	1.54253433
CDC25B	0	0	0	1.5407365
ASTE1	0	0	0	1.54072184
FANCL	0	0	0	1.54059533
GLUL	0	0	0	1.54057029
NFATC1	0	0	0	1.54002935
HLX	0	0	0	1.53990829
MAD2L1	0	0	0	1.53983885
CCL13	0	0	0	1.53979585
WASF2	0	0	0	1.53892004
ATG12	0	0	0	1.5387196
GALNT6	0	0	0	1.5381675
MELK	0	0	0	1.53744022
CHORDC1	0	0	0	1.53732117
KIF2C	0	0	0	1.53660845
SPRY1	0	0	0	1.53635951
NPAT	0	0	0	1.53602363
LIPA	0	0	0	1.53550836

© 2021 Rychkov D et al. *JAMA* Network Open.

APBA2	0	0	0	1.53504876
FHL2	0	0	0	1.53438862
QPCT	0	0	0	1.53300864
CXCL6	0	0	0	1.53275773
EDN1	0	0	0	1.53248591
LHFPL2	0	0	0	1.53166749
NXPE3	0	0	0	1.53084475
IL6	0	0	0	1.52863482
SMC4	0	0	0	1.52786386
CNOT2	0	0	0	1.52785618
MX1	0	0	0	1.52736379
COL4A1	0	0	0	1.52675306
INPP4A	0	0	0	1.52644134
INHBA	0	0	0	1.52618051
MYOF	0	0	0	1.52614095
FAS	0	0	0	1.52599056
H1FX	0	0	0	1.52530175
VRK2	0	0	0	1.5252127
ARGLU1	0	0	0	1.52478537
FRAT2	0	0	0	1.5245678
NFE2L3	0	0	0	1.5244434
IL12A	0	0	0	1.52442253
CYBC1	0	0	0	1.52402704
ARID5A	0	0	0	1.5234248
FBN2	0	0	0	1.5221886
CALHM2	0	0	0	1.52215745
CCNB2	0	0	0	1.52193922
SPHK1	0	0	0	1.52151297
MAP7D1	0	0	0	1.52136532

© 2021 Rychkov D et al. *JAMA* Network Open.

ABCA7	0	0	0	1.52071519
FBXW7	0	0	0	1.52029374
TOPBP1	0	0	0	1.51999377
EFEMP1	0	0	0	1.51988885
LBR	0	0	0	1.51849527
FNBP4	0	0	0	1.51786188
CHI3L2	0	0	0	1.5177083
CDK1	0	0	0	1.5176316
RARRES3	0	0	0	1.51611087
KCNMA1	0	0	0	1.51594839
TICAM1	0	0	0	1.51481621
GRB2	0	0	0	1.51462834
MYCBP2	0	0	0	1.51459376
TM6SF1	0	0	0	1.51401445
CENPE	0	0	0	1.51383525
TSPAN13	0	0	0	1.51264487
BCAT1	0	0	0	1.51257381
STAT5A	0	0	0	1.51250663
CCNB1	0	0	0	1.5118339
MCM5	0	0	0	1.51143735
DENND5A	0	0	0	1.51094099
ZNF211	0	0	0	1.51070651
BMP2K	0	0	0	1.50972453
ARHGAP17	0	0	0	1.5085973
NEDD9	0	0	0	1.50832738
DEGS1	0	0	0	1.50804227
OSER1	0	0	0	1.5078846
APOBEC3F	0	0	0	1.50788158
CPVL	0	0	0	1.50718093

© 2021 Rychkov D et al. *JAMA* Network Open.

ITPKC	0	0	0	1.50618707
LHFPL6	0	0	0	1.50449833
NAP1L1	0	0	0	1.50424811
IL4R	0	0	0	1.50413413
C5AR1	0	0	0	1.50355445
PAK3	0	0	0	1.50271748
BUB1B	0	0	0	1.50206433
HIVEP3	0	0	0	1.50116617
CD14	0	0	0	1.50105813
WDR82	0	0	0	1.50040446
ABCC1	0	0	0	1.50023789

© 2021 Rychkov D et al. *JAMA* Network Open.

eTable 2b. Downregulated Differentially Expressed Genes From SAM Analysis of Comparison of Acute Rejection to Normal Kidney Tissues

gene	raw p.value	q.value	Bonferroni adj. p-value	fold change
KLK1	0	0	0	0.48693773
GOT1	0	0	0	0.49355899
PTGER3	0	0	0	0.4943702
PVALB	0	0	0	0.49798708
HYAL1	0	0	0	0.50742891
EPB41L5	0	0	0	0.51470652
DARS2	0	0	0	0.51750457
NNT	0	0	0	0.51877222
FGF1	0	0	0	0.52043111
MAGI2	0	0	0	0.52166555
CERS2	0	0	0	0.52282638
PTPN3	0	0	0	0.52743075
MOCS1	0	0	0	0.52829329
NEDD4L	0	0	0	0.52829374
GAD1	0	0	0	0.52831202
RHCG	0	0	0	0.53137225
KBTBD11	0	0	0	0.53250661
HADH	0	0	0	0.53293268
SGK2	0	0	0	0.53511649
FCN3	0	0	0	0.53743944
DLAT	0	0	0	0.53832852
KLK7	0	0	0	0.53880302
DNAJA3	0	0	0	0.54192505
LRPPRC	0	0	0	0.54362972
EPHX1	0	0	0	0.54501674

© 2021 Rychkov D et al. *JAMA* Network Open.

GRB10	0	0	0	0.54524437
DMTN	0	0	0	0.54590412
FOXI1	0	0	0	0.5459359
SMPD1	0	0	0	0.54642479
HSPA9	0	0	0	0.54762147
PHB	0	0	0	0.54820387
TMEM177	0	0	0	0.54909008
GPD1L	0	0	0	0.54963689
DHDDS	0	0	0	0.54968704
ATP6V1H	0	0	0	0.5501728
SPOCK1	0	0	0	0.5509999
SLC4A1	0	0	0	0.55186191
GHR	0	0	0	0.5528509
BCL2L13	0	0	0	0.55294053
TMEM59	0	0	0	0.55513674
DPP6	0	0	0	0.55538654
B4GAT1	0	0	0	0.55817151
CRISP2	0	0	0	0.55921448
CRHBP	0	0	0	0.55942343
HMGCS2	0	0	0	0.56031121
ADTRP	0	0	0	0.56037491
SLC25A16	0	0	0	0.5603992
AGAP1	0	0	0	0.56158299
NECAB3	0	0	0	0.56204669
SPR	0	0	0	0.56226431
SYT13	0	0	0	0.56245316
RPUSD2	0	0	0	0.56256769
BCL2L2	0	0	0	0.5625695
AQP6	0	0	0	0.5632812

© 2021 Rychkov D et al. *JAMA* Network Open.

GRPEL1	0	0	0	0.56398692
SELENBP1	0	0	0	0.56420905
AMFR	0	0	0	0.56512681
ST7	0	0	0	0.56621966
BMP7	0	0	0	0.56669982
PLA2R1	0	0	0	0.56698494
AK2	0	0	0	0.56707115
METTL1	0	0	0	0.56770595
SEMA4G	0	0	0	0.56804585
MPP5	0	0	0	0.56841029
MDH2	0	0	0	0.56865825
TSFM	0	0	0	0.56886242
COQ3	0	0	0	0.57009015
TEX2	0	0	0	0.57081985
CYP2B6	0	0	0	0.57109868
RHBG	0	0	0	0.57141713
PATJ	0	0	0	0.57151787
DERA	0	0	0	0.57168891
EEF1AKMT3	0	0	0	0.57185939
UNC13B	0	0	0	0.57238605
ETFDH	0	0	0	0.57250322
ALKBH4	0	0	0	0.572826
SUCLG1	0	0	0	0.57333157
SDC1	0	0	0	0.57336569
MAOA	0	0	0	0.57338253
USP46	0	0	0	0.57369374
ADGRF5	0	0	0	0.57378664
CA2	0	0	0	0.57380655
TNNT2	0	0	0	0.5739987

© 2021 Rychkov D et al. *JAMA* Network Open.

ERAL1	0	0	0	0.57514697
CLDN8	0	0	0	0.57563316
TMEM53	0	0	0	0.57578417
RORC	0	0	0	0.57584417
NDUFS1	0	0	0	0.57585491
GOT2	0	0	0	0.5759408
RAB17	0	0	0	0.57597645
CYB5R1	0	0	0	0.57603037
AHCYL2	0	0	0	0.57637154
MACROD1	0	0	0	0.5767688
NCS1	0	0	0	0.57732837
POMGNT1	0	0	0	0.57739651
KCNJ3	0	0	0	0.577664
EGF	0	0	0	0.577745
TACO1	0	0	0	0.57782508
CTDSPL	0	0	0	0.57785348
PLCE1	0	0	0	0.57810271
TLN2	0	0	0	0.5782955
GATB	0	0	0	0.57945595
PCP4	0	0	0	0.57954602
INSR	0	0	0	0.57960972
ESRRG	0	0	0	0.57976357
ACADL	0	0	0	0.57985369
CNNM2	0	0	0	0.58047182
EBP	0	0	0	0.58060167
CHUK	0	0	0	0.58073876
SERPINA5	0	0	0	0.58101498
COASY	0	0	0	0.58149283
KCNK10	0	0	0	0.58167768

© 2021 Rychkov D et al. *JAMA* Network Open.

SLC17A5	0	0	0	0.582197
PRRG2	0	0	0	0.58247622
ACADSB	0	0	0	0.58303886
TCTA	0	0	0	0.58311468
ATRN	0	0	0	0.58319989
NDNF	0	0	0	0.58338802
ALAD	0	0	0	0.58369689
SLC28A1	0	0	0	0.58384042
SPACA9	0	0	0	0.58413017
SLC25A11	0	0	0	0.58434323
ATG4A	0	0	0	0.58486939
SAMM50	0	0	0	0.58530405
RMDN3	0	0	0	0.585969
PNPO	0	0	0	0.58609304
HADHA	0	0	0	0.58627052
C11orf71	0	0	0	0.58647431
SLC39A7	0	0	0	0.58674849
ALB	0	0	0	0.58676306
SLC16A1	0	0	0	0.5868951
PEX5	0	0	0	0.58709234
SPAG5	0	0	0	0.58711065
SLC35A2	0	0	0	0.58726177
ARHGAP28	0	0	0	0.58783024
GHITM	0	0	0	0.58802154
DIP2C	0	0	0	0.5881149
VEGFA	0	0	0	0.5882683
AGBL5	0	0	0	0.58917221
PLEKHA6	0	0	0	0.58933098
HAGH	0	0	0	0.58997309

© 2021 Rychkov D et al. *JAMA* Network Open.

GPHN	0	0	0	0.59001746
CCDC106	0	0	0	0.5903418
RNF123	0	0	0	0.59034663
THRA	0	0	0	0.59050606
IDH3A	0	0	0	0.5905642
YIPF6	0	0	0	0.59109059
PDHA1	0	0	0	0.5915313
SLC25A4	0	0	0	0.59177679
MME	0	0	0	0.59211399
TRPM6	0	0	0	0.59231096
ANXA9	0	0	0	0.59245628
FBXO3	0	0	0	0.59250347
PREPL	0	0	0	0.59276339
SLC13A3	0	0	0	0.59291266
AP1M2	0	0	0	0.59312721
DHCR7	0	0	0	0.59317922
KIF16B	0	0	0	0.59333547
APOO	0	0	0	0.59343205
ATP6V1D	0	0	0	0.59345771
DUS4L	0	0	0	0.59360866
THRB	0	0	0	0.59372501
IDH3B	0	0	0	0.5939076
TBC1D13	0	0	0	0.59435672
MYH14	0	0	0	0.59442735
ATP5MC3	0	0	0	0.59461173
RGS7	0	0	0	0.59548685
TXN2	0	0	0	0.59604469
ATP6V0A4	0	0	0	0.59606826
PFKFB2	0	0	0	0.59620238

© 2021 Rychkov D et al. *JAMA* Network Open.

ACOT11	0	0	0	0.59633406
PRKCA	0	0	0	0.59653172
LARP4	0	0	0	0.59678552
CFAP410	0	0	0	0.59710483
FABP1	0	0	0	0.59711694
MIEF1	0	0	0	0.59742851
ST6GALNAC2	0	0	0	0.59772809
GABARAPL1	0	0	0	0.5984235
ESRRA	0	0	0	0.59848421
THEM6	0	0	0	0.59851838
HIRIP3	0	0	0	0.59920123
LIN7A	0	0	0	0.59945822
RPRD1A	0	0	0	0.5995077
TMPRSS2	0	0	0	0.60050676
RBBP9	0	0	0	0.60057405
SORT1	0	0	0	0.60084207
PHLDA1	0	0	0	0.60109607
MCCC1	0	0	0	0.60122329
ACOX1	0	0	0	0.6022099
TM9SF4	0	0	0	0.60260206
SERPINA4	0	0	0	0.6027118
COA3	0	0	0	0.6027593
MIPEP	0	0	0	0.6030818
PCBP2	0	0	0	0.60314059
TFB2M	0	0	0	0.60318532
NAXE	0	0	0	0.60344618
RRAGD	0	0	0	0.60364212
NDUFS3	0	0	0	0.60421724
ANO10	0	0	0	0.60443645

© 2021 Rychkov D et al. *JAMA* Network Open.

INPP5J	0	0	0	0.60461323
ANKRD46	0	0	0	0.60489972
CKMT2	0	0	0	0.60504564
SLC12A3	0	0	0	0.6052145
SLC19A2	0	0	0	0.60525228
MAIP1	0	0	0	0.60526195
IPO13	0	0	0	0.60551276
NDUFAF4	0	0	0	0.60562692
SNTA1	0	0	0	0.60630246
COPS6	0	0	0	0.60630325
SLC12A2	0	0	0	0.60630451
CLPB	0	0	0	0.60642462
MAPT	0	0	0	0.60673555
CEL	0	0	0	0.60677796
F8	0	0	0	0.60709187
GAS2	0	0	0	0.60744796
ILVBL	0	0	0	0.60747335
MRPL48	0	0	0	0.60752603
NT5DC2	0	0	0	0.60772066
ADIPOR1	0	0	0	0.60773454
ABCC2	0	0	0	0.60773882
PAFAH2	0	0	0	0.60808396
NFS1	0	0	0	0.60813229
ZC3H7B	0	0	0	0.6088623
NECAB2	0	0	0	0.60905156
CRYL1	0	0	0	0.60905185
TMEM115	0	0	0	0.60961812
PEX26	0	0	0	0.61020148
WWOX	0	0	0	0.61042824

© 2021 Rychkov D et al. *JAMA* Network Open.

EXOSC7	0	0	0	0.61048453
RSAD1	0	0	0	0.61063273
PHKA2	0	0	0	0.61093552
ESRP2	0	0	0	0.61094959
CDADC1	0	0	0	0.61098197
VPS13D	0	0	0	0.61110758
TEX261	0	0	0	0.61122808
PCCB	0	0	0	0.61139246
PLOD1	0	0	0	0.6113975
DMAC2	0	0	0	0.61145839
PGM1	0	0	0	0.61178904
FBXO22	0	0	0	0.61207341
FAAH	0	0	0	0.61236188
ATP6V0A1	0	0	0	0.61250224
HDAC11	0	0	0	0.61268633
C16orf58	0	0	0	0.61270468
SLIT2	0	0	0	0.61275685
SIRT5	0	0	0	0.61288669
PLPPR1	0	0	0	0.61319815
APOOL	0	0	0	0.61320403
EYA2	0	0	0	0.61328219
MRPL2	0	0	0	0.61331547
PIGV	0	0	0	0.61372264
ENTPD5	0	0	0	0.61395718
SPHK2	0	0	0	0.6139609
SMO	0	0	0	0.61414674
MRPS22	0	0	0	0.61462259
COQ9	0	0	0	0.61465797
RBKS	0	0	0	0.61509214

© 2021 Rychkov D et al. *JAMA* Network Open.

SIRT4	0	0	0	0.61534091
TGFBR3	0	0	0	0.6153716
FASTKD5	0	0	0	0.61554387
FDX1	0	0	0	0.61569514
ERLIN2	0	0	0	0.61591158
MT3	0	0	0	0.61609144
NDUFS2	0	0	0	0.61623592
PGRMC2	0	0	0	0.61629667
ALDH1L1	0	0	0	0.61709833
TUBGCP3	0	0	0	0.61722159
ECHDC3	0	0	0	0.61727769
SLC22A4	0	0	0	0.61755181
ANGPTL3	0	0	0	0.6176852
DNAJC6	0	0	0	0.61782934
GLRX5	0	0	0	0.61783796
CREM	0	0	0	0.61790933
ASL	0	0	0	0.61806758
PC	0	0	0	0.61819734
GJA5	0	0	0	0.61834751
GJA3	0	0	0	0.61894702
TCTN3	0	0	0	0.61903001
NPHS2	0	0	0	0.61920176
SH3GL2	0	0	0	0.61934173
DHRS11	0	0	0	0.6193877
ST3GAL6	0	0	0	0.61938787
SUOX	0	0	0	0.6195785
PEX7	0	0	0	0.62008356
ALG8	0	0	0	0.62029325
MPDU1	0	0	0	0.62039811

© 2021 Rychkov D et al. *JAMA* Network Open.

PDSS2	0	0	0	0.62058058
COBL	0	0	0	0.62064794
MLYCD	0	0	0	0.62068419
FBXO17	0	0	0	0.62070449
CALB1	0	0	0	0.62084487
CDK18	0	0	0	0.62094492
FBXO21	0	0	0	0.62100758
NRSN2	0	0	0	0.62112565
PLG	0	0	0	0.62121514
TPD52	0	0	0	0.62137016
MRPS15	0	0	0	0.62152315
ATP6AP1	0	0	0	0.62154654
RITA1	0	0	0	0.6216024
C1orf56	0	0	0	0.62173459
RASSF8	0	0	0	0.62179112
SDHB	0	0	0	0.62179538
PACRG	0	0	0	0.6219101
FH	0	0	0	0.62191451
DDX25	0	0	0	0.62199322
ALS2CL	0	0	0	0.62201975
ADCY1	0	0	0	0.62314047
ETNK2	0	0	0	0.62345784
PCOLCE2	0	0	0	0.62397057
MTFR1	0	0	0	0.62411514
NAGLU	0	0	0	0.62426877
NSDHL	0	0	0	0.62434014
SEMA3B	0	0	0	0.62439207
SLC12A6	0	0	0	0.62439855
FABP3	0	0	0	0.62449417

© 2021 Rychkov D et al. *JAMA* Network Open.

EXPH5	0	0	0	0.62497022
ATP6V0E2	0	0	0	0.62499653
LONRF3	0	0	0	0.62528715
ACAD8	0	0	0	0.62536974
EPM2A	0	0	0	0.62545119
AHCYL1	0	0	0	0.62557471
ATP6V1E1	0	0	0	0.62593565
CDKL1	0	0	0	0.62600789
IL1RL1	0	0	0	0.62619322
RABGGTB	0	0	0	0.62621477
HSD11B2	0	0	0	0.62629477
KDR	0	0	0	0.62652338
AMT	0	0	0	0.62682084
CLCN6	0	0	0	0.62695807
RDH11	0	0	0	0.62699175
TBCE	0	0	0	0.62714463
CALM1	0	0	0	0.62717451
SIK3	0	0	0	0.62741656
EMX1	0	0	0	0.62757593
DNASE1	0	0	0	0.6277006
MAPKAP1	0	0	0	0.62807001
TOM1L2	0	0	0	0.6281099
CAT	0	0	0	0.62821053
ABHD10	0	0	0	0.62830921
SNX4	0	0	0	0.62838296
ABLIM3	0	0	0	0.6284581
KYAT1	0	0	0	0.62865012
MFAP3L	0	0	0	0.62906265
RNF14	0	0	0	0.62910385

© 2021 Rychkov D et al. *JAMA* Network Open.

DEXI	0	0	0	0.62916512
TAF7L	0	0	0	0.62924244
TM7SF2	0	0	0	0.62928578
RPAP1	0	0	0	0.62944379
CYC1	0	0	0	0.62948498
OSBP2	0	0	0	0.62971168
EMCN	0	0	0	0.62974627
ABHD6	0	0	0	0.62979186
PTPRO	0	0	0	0.63010676
ZHX3	0	0	0	0.63041814
MAOB	0	0	0	0.63047255
LPCAT3	0	0	0	0.63076105
CD320	0	0	0	0.63077301
UQCRC1	0	0	0	0.63081985
ARHGEF12	0	0	0	0.63103287
PSME3	0	0	0	0.63104238
LDHB	0	0	0	0.63120793
ALDH1B1	0	0	0	0.63133618
HPN	0	0	0	0.63137053
AIFM1	0	0	0	0.63171872
NDUFS4	0	0	0	0.63172519
CORO2B	0	0	0	0.63181432
ZDHHC3	0	0	0	0.63188173
PDK2	0	0	0	0.63192757
NDUFB8	0	0	0	0.63206378
APEH	0	0	0	0.63212384
GLUD2	0	0	0	0.63221469
NDUFS8	0	0	0	0.6323382
RNF128	0	0	0	0.63238062

© 2021 Rychkov D et al. *JAMA* Network Open.

SAP18	0	0	0	0.63256985
EPN3	0	0	0	0.63272262
COX10	0	0	0	0.63278903
PRSS8	0	0	0	0.63298935
SSTR2	0	0	0	0.63329916
PEX19	0	0	0	0.63336093
SULT1C2	0	0	0	0.63336163
AVPR1A	0	0	0	0.63337304
MAP7	0	0	0	0.6336401
COQ6	0	0	0	0.63376709
PLD1	0	0	0	0.63399403
CCDC25	0	0	0	0.63405646
TOM1	0	0	0	0.63408286
OGDH	0	0	0	0.63415406
SC5D	0	0	0	0.63416204
GCNT2	0	0	0	0.6341973
MDH1	0	0	0	0.63435781
ARHGEF28	0	0	0	0.63461475
ABCA5	0	0	0	0.63467778
SIGMAR1	0	0	0	0.63475993
ATP5F1B	0	0	0	0.63477416
ACAT1	0	0	0	0.63481679
HSDL2	0	0	0	0.63496993
SHANK2	0	0	0	0.6351465
MINDY1	0	0	0	0.63526891
MRPS35	0	0	0	0.63528374
STAP1	0	0	0	0.63532722
CDK16	0	0	0	0.63547831
ACO2	0	0	0	0.63572218

© 2021 Rychkov D et al. *JAMA* Network Open.

ZER1	0	0	0	0.63585672
ACAT2	0	0	0	0.63595498
ACSS3	0	0	0	0.63628514
PXMP4	0	0	0	0.63641961
WDR59	0	0	0	0.63645301
PANK3	0	0	0	0.63646901
STAC	0	0	0	0.63649309
SMUG1	0	0	0	0.63661915
ACO1	0	0	0	0.63680922
ASB9	0	0	0	0.63688872
SLC39A4	0	0	0	0.63703726
BDH1	0	0	0	0.63708972
NDUFA9	0	0	0	0.63711
MPC2	0	0	0	0.63711689
PPARA	0	0	0	0.637132
IGF1R	0	0	0	0.63728971
ARL2	0	0	0	0.63755803
DOLK	0	0	0	0.63758921
EFHD1	0	0	0	0.63775323
BNIP3	0	0	0	0.63817766
CWH43	0	0	0	0.6383168
NARS2	0	0	0	0.63832098
ARHGEF17	0	0	0	0.63897513
APLP1	0	0	0	0.638999
CLIC5	0	0	0	0.63901431
SLC4A2	0	0	0	0.6390521
SIRT3	0	0	0	0.63911534
PRKAG2	0	0	0	0.63953909
SCN9A	0	0	0	0.63960949

© 2021 Rychkov D et al. *JAMA* Network Open.

CTH	0	0	0	0.63992852
NDST1	0	0	0	0.640028
ATP1B2	0	0	0	0.64031619
ROGDI	0	0	0	0.64070625
TOLLIP	0	0	0	0.64085755
VTI1B	0	0	0	0.64101587
LRPAP1	0	0	0	0.64115832
NELL1	0	0	0	0.64128702
CYFIP2	0	0	0	0.64143041
SLC16A10	0	0	0	0.64145366
ATP5MC1	0	0	0	0.64145609
AIMP2	0	0	0	0.6414617
ASB8	0	0	0	0.6416399
TXNRD2	0	0	0	0.64167039
PRKCZ	0	0	0	0.64177746
UFSP2	0	0	0	0.64186357
NR2F6	0	0	0	0.64196695
GRB14	0	0	0	0.64211677
EXD2	0	0	0	0.64218993
HPGD	0	0	0	0.64218996
POLR3B	0	0	0	0.64237098
PDE1C	0	0	0	0.64249782
ARL15	0	0	0	0.64250653
SCNN1B	0	0	0	0.6425776
DNAJC11	0	0	0	0.64258478
CPT2	0	0	0	0.64266474
CYP46A1	0	0	0	0.64318176
CKB	0	0	0	0.64318991
SSX2IP	0	0	0	0.64336309

© 2021 Rychkov D et al. *JAMA* Network Open.

ARFIP2	0	0	0	0.64341072
SLC22A6	0	0	0	0.64346011
TMBIM6	0	0	0	0.64346594
GALT	0	0	0	0.64376495
PEPD	0	0	0	0.64385641
PKLR	0	0	0	0.64396659
PSEN2	0	0	0	0.64398445
TSG101	0	0	0	0.64407375
UQCR10	0	0	0	0.64409706
DLD	0	0	0	0.64412753
ST8SIA5	0	0	0	0.64413894
SPRYD7	0	0	0	0.64416525
DNAJA2	0	0	0	0.64417773
SPINK1	0	0	0	0.64429495
SFXN1	0	0	0	0.64433592
SLC37A4	0	0	0	0.64433648
ACTR1B	0	0	0	0.64440448
FGFBP1	0	0	0	0.64445276
FAH	0	0	0	0.64451502
NEBL	0	0	0	0.64461942
PAICS	0	0	0	0.64490971
CHI3L1	0	0	0	0.64505794
MOCS2	0	0	0	0.64522849
GSS	0	0	0	0.64536848
PDE8A	0	0	0	0.64552791
PEX10	0	0	0	0.64596534
CYP17A1	0	0	0	0.64604305
UQCC1	0	0	0	0.64618577
MINPP1	0	0	0	0.64635729

© 2021 Rychkov D et al. *JAMA* Network Open.

PLEKHB1	0	0	0	0.64648183
SLC35A3	0	0	0	0.64664369
RAB11FIP3	0	0	0	0.6467728
POMT1	0	0	0	0.64682848
GSTM4	0	0	0	0.64691523
ME3	0	0	0	0.64708134
CLN8	0	0	0	0.64738334
MARC1	0	0	0	0.64741742
NR4A1	0	0	0	0.64749709
BTD	0	0	0	0.64801782
SNX27	0	0	0	0.64809249
OSBPL1A	0	0	0	0.64834266
PMVK	0	0	0	0.64837156
AQP3	0	0	0	0.64849579
MFN2	0	0	0	0.64850579
NUDT6	0	0	0	0.64858955
IMPA2	0	0	0	0.6486293
ENDOG	0	0	0	0.64867917
ECSIT	0	0	0	0.64868609
OCEL1	0	0	0	0.64868645
ST18	0	0	0	0.64869864
MRPS2	0	0	0	0.6487683
MPC1	0	0	0	0.6487699
EPB41L4B	0	0	0	0.64881767
PFKM	0	0	0	0.64889656
EHBP1	0	0	0	0.64893805
UPB1	0	0	0	0.64895151
SLC5A6	0	0	0	0.64895577
TUBAL3	0	0	0	0.64907405

© 2021 Rychkov D et al. *JAMA* Network Open.

CCNB1IP1	0	0	0	0.64910631
SLC35D1	0	0	0	0.64927318
MKKS	0	0	0	0.64936794
RAP1GAP	0	0	0	0.64952879
PAK4	0	0	0	0.64956086
PEX3	0	0	0	0.64961022
RMND5B	0	0	0	0.64974056
PDZD8	0	0	0	0.65009089
C1orf115	0	0	0	0.65015079
FA2H	0	0	0	0.65028245
ADGRF1	0	0	0	0.65030081
ATP6V1C1	0	0	0	0.65030632
ATP5F1A	0	0	0	0.65081715
CLMN	0	0	0	0.65087878
AHCY	0	0	0	0.65093237
MARC2	0	0	0	0.65103607
PLEKHA5	0	0	0	0.6512294
NDRG2	0	0	0	0.65132383
SDHAF3	0	0	0	0.65154858
NPHS1	0	0	0	0.65167196
ADCY9	0	0	0	0.65192817
CLPTM1	0	0	0	0.65199348
RALYL	0	0	0	0.65214836
ANK2	0	0	0	0.652229
ACAA1	0	0	0	0.65227288
GCDH	0	0	0	0.65234325
ADH5	0	0	0	0.65259713
CDIP1	0	0	0	0.65261939
TSPAN7	0	0	0	0.65274959

© 2021 Rychkov D et al. *JAMA* Network Open.

LMBRD1	0	0	0	0.65279721
TEX264	0	0	0	0.65285213
SHARPIN	0	0	0	0.65351209
CIDEB	0	0	0	0.65354068
HAO1	0	0	0	0.65365529
SETMAR	0	0	0	0.65397413
SLC25A10	0	0	0	0.65399028
RAB3A	0	0	0	0.65401716
SLC7A10	0	0	0	0.65416239
TPMT	0	0	0	0.65418989
HRG	0	0	0	0.65455769
CACNA1D	0	0	0	0.65481452
EMC1	0	0	0	0.65490098
INKA2	0	0	0	0.65491706
CISD1	0	0	0	0.65549494
MRPS7	0	0	0	0.65550108
PHF7	0	0	0	0.65577024
ABCB6	0	0	0	0.65612452
GRTP1	0	0	0	0.65619041
TFAP2A	0	0	0	0.65632531
STUB1	0	0	0	0.65643208
ATP1A1	0	0	0	0.65670569
GRHPR	0	0	0	0.65672816
SUCLG2	0	0	0	0.65675561
FOLR3	0	0	0	0.65689424
GPR137	0	0	0	0.6571419
ATP6V1B1	0	0	0	0.65718845
ETFB	0	0	0	0.65739331
PHYH	0	0	0	0.65748304

© 2021 Rychkov D et al. *JAMA* Network Open.

ATXN7L1	0	0	0	0.65752238
SCAP	0	0	0	0.65765304
DLST	0	0	0	0.65771883
KIAA0232	0	0	0	0.65773484
PCBD1	0	0	0	0.65777665
SLC26A4	0	0	0	0.65794581
WBP2	0	0	0	0.65794832
TACC2	0	0	0	0.65795819
CHCHD3	0	0	0	0.65809824
POLR2C	0	0	0	0.65821282
CRYZ	0	0	0	0.65823033
MAB21L4	0	0	0	0.65825044
PPIF	0	0	0	0.65838071
AKAP1	0	0	0	0.65841778
HTRA1	0	0	0	0.65853041
CYB5A	0	0	0	0.65866192
PEBP1	0	0	0	0.65869036
ZGPAT	0	0	0	0.65873034
ALLC	0	0	0	0.65892579
ITPR1	0	0	0	0.65900321
ATP5F1C	0	0	0	0.6590526
RAB11FIP5	0	0	0	0.65913982
MAGI1	0	0	0	0.65927579
ATP6V1G2	0	0	0	0.65936406
KIAA0391	0	0	0	0.6598485
SHC3	0	0	0	0.66020766
MCOLN3	0	0	0	0.66046096
UAP1	0	0	0	0.66058953
USP5	0	0	0	0.66065441

© 2021 Rychkov D et al. *JAMA* Network Open.

PDZRN3	0	0	0	0.66088434
VPS26C	0	0	0	0.66091172
CBR1	0	0	0	0.66130666
MARK1	0	0	0	0.66132662
TRPM3	0	0	0	0.6613496
MCAT	0	0	0	0.66143952
MSRA	0	0	0	0.6615252
ZCCHC14	0	0	0	0.66159982
VAC14	0	0	0	0.66172644
FASTKD2	0	0	0	0.66176906
AP3B1	0	0	0	0.66193913
UBOX5	0	0	0	0.66196981
DUSP3	0	0	0	0.66197201
SLC3A2	0	0	0	0.6619813
ZSCAN5A	0	0	0	0.66226148
DCTN1	0	0	0	0.66246816
ADI1	0	0	0	0.66254295
DLEU1	0	0	0	0.66260859
FHIT	0	0	0	0.66266209
ARHGAP24	0	0	0	0.66269943
FAM50B	0	0	0	0.66289696
THAP9	0	0	0	0.66305098
CES2	0	0	0	0.66324575
GRAMD1C	0	0	0	0.66325097
PSMD9	0	0	0	0.6632865
SCNN1A	0	0	0	0.66340933
PRLR	0	0	0	0.66341194
ALDH6A1	0	0	0	0.66346541
GPS2	0	0	0	0.66350097

© 2021 Rychkov D et al. *JAMA* Network Open.

PLA2G12A	0	0	0	0.6635572
AUH	0	0	0	0.66361983
ELAVL1	0	0	0	0.66401828
HDDC2	0	0	0	0.66405147
ASB13	0	0	0	0.66421514
TP53TG5	0	0	0	0.66441591
TMEM94	0	0	0	0.6645981
SLC26A6	0	0	0	0.66462376
DLGAP2	0	0	0	0.664645
ICMT	0	0	0	0.66466051
DCXR	0	0	0	0.66503721
UROD	0	0	0	0.66503874
UQCRC2	0	0	0	0.66510283
MRPL42	0	0	0	0.66532017
EHD3	0	0	0	0.6654402
MICU1	0	0	0	0.6656005
MRPS18B	0	0	0	0.66579376
LARP6	0	0	0	0.66587277
PCTP	0	0	0	0.66589734
HMOX2	0	0	0	0.66591241
SLC1A1	0	0	0	0.66592445
NPAS2	0	0	0	0.66605708
ZC2HC1C	0	0	0	0.66637196
WDTC1	0	0	0	0.66639081
SLC7A8	0	0	0	0.66647586
SDHD	0	0	0	0.66663406
UBE2D4	0	0	0	0.66665164

© 2021 Rychkov D et al. *JAMA* Network Open.

eTable 3. Cell Types Considered in Cell Type Enrichment Analysis With xCell

Cells	Family	Type
Erythrocytes	HSC	HSC
HSC	HSC	HSC
Megakaryocytes	HSC	HSC
Platelets	HSC	HSC
B-cells	Immune	Lymphoid
CD4+ memory T-cells	Immune	Lymphoid
CD4+ naive T-cells	Immune	Lymphoid
CD4+ T-cells	Immune	Lymphoid
CD4+ Tcm	Immune	Lymphoid
CD4+ Tem	Immune	Lymphoid
CD8+ naive T-cells	Immune	Lymphoid
CD8+ T-cells	Immune	Lymphoid
CD8+ Tcm	Immune	Lymphoid
CD8+ Tem	Immune	Lymphoid
Class-switched memory B-cells	Immune	Lymphoid
Memory B-cells	Immune	Lymphoid
naive B-cells	Immune	Lymphoid
NK cells	Immune	Lymphoid
NKT	Immune	Lymphoid
Plasma cells	Immune	Lymphoid
pro B-cells	Immune	Lymphoid
Tgd cells	Immune	Lymphoid
Th1 cells	Immune	Lymphoid
Th2 cells	Immune	Lymphoid
Tregs	Immune	Lymphoid
aDC	Immune	Myeloid
Basophils	Immune	Myeloid

© 2021 Rychkov D et al. *JAMA* Network Open.

cDC	Immune	Myeloid
DC	Immune	Myeloid
Eosinophils	Immune	Myeloid
iDC	Immune	Myeloid
Macrophages	Immune	Myeloid
Macrophages M1	Immune	Myeloid
Macrophages M2	Immune	Myeloid
Mast cells	Immune	Myeloid
Monocytes	Immune	Myeloid
Neutrophils	Immune	Myeloid
pDC	Immune	Myeloid
Epithelial cells	Non-Hematopoietic	Epithelial
Endothelial cells	Non-Hematopoietic	Stroma
Fibroblasts	Non-Hematopoietic	Stroma
ly Endothelial cells	Non-Hematopoietic	Stroma
Mesangial cells	Non-Hematopoietic	Stroma
MSC	Non-Hematopoietic	Stroma
mv Endothelial cells	Non-Hematopoietic	Stroma

eReferences

1. Barrett T, Wilhite SE, Ledoux P, et al. NCBI GEO: Archive for functional genomics data sets - Update. *Nucleic Acids Res*. 2013;41(D1):991-995. doi:10.1093/nar/gks1193

2. Waldron L, Riester M, Ramos M, Parmigiani G, Birrer M. The doppelganger effect: Hidden duplicates in databases of transcriptome profiles. *J Natl Cancer Inst*. 2016;108(11):2-5. doi:10.1093/jnci/djw146

3. R core team. R: A language and environment for statistical computing. *R Found Stat Comput Vienna, Austria*. 2018. http://www.r-project.org/.

4. Piccolo SR, Sun Y, Campbell JD, Lenburg ME, Bild AH, Johnson WE. A single-sample microarray normalization method to facilitate personalized-medicine workflows. *Genomics*. 2012;100(6):337-344. doi:10.1016/j.ygeno.2012.08.003

5. Huber W, Carey VJ, Gentleman R, et al. Orchestrating high-throughput genomic analysis with Bioconductor. *Nat Methods*. 2015;12(2):115-121. doi:10.1038/nmeth.3252

© 2021 Rychkov D et al. *JAMA* Network Open.

6. Irizarry RA. Exploration, normalization, and summaries of high density oligonucleotide array probe level data. *Biostatistics*. 2003;4(2):249-264. doi:10.1093/biostatistics/4.2.249

7. Dai M, Wang P, Boyd AD, et al. Evolving gene/transcript definitions significantly alter the interpretation of GeneChip data. *Nucleic Acids Res*. 2005;33(20):e175-e175. doi:10.1093/nar/gni179

8. Ritchie ME, Phipson B, Wu D, et al. limma powers differential expression analyses for RNA-sequencing and microarray studies. *Nucleic Acids Res*. 2015;43(7):e47-e47. doi:10.1093/nar/gkv007

9. Shi W, Oshlack A, Smyth GK. Optimizing the noise versus bias trade-off for Illumina whole genome expression BeadChips. *Nucleic Acids Res*. 2010;38(22). doi:10.1093/nar/gkq871

10. Durinck S, Spellman PT, Birney E, Huber W. Mapping identifiers for the integration of genomic datasets with the R/ Bioconductor package biomaRt. *Nat Protoc*. 2009;4(8):1184-1191. doi:10.1038/nprot.2009.97

11. Leek JT, Scharpf RB, Bravo HC, et al. Tackling the widespread and critical impact of batch effects in high-throughput data. *Nat Rev Genet*. 2010;11(10):733-739. doi:10.1038/nrg2825

12. Jaffe AE, Hyde T, Kleinman J, et al. Practical impacts of genomic data "cleaning" on biological discovery using surrogate variable analysis. *BMC Bioinformatics*. 2015;16(1):372. doi:10.1186/s12859-015-0808-5

13. Goh WW Bin, Wang W, Wong L. Why Batch Effects Matter in Omics Data, and How to Avoid Them. *Trends Biotechnol*. 2017;35(6):498-507. doi:10.1016/j.tibtech.2017.02.012

14. Lazar C, Meganck S, Taminau J, et al. Batch effect removal methods for microarray gene expression data integration: a survey. *Brief Bioinform*. 2013;14(4):469-490. doi:10.1093/bib/bbs037

15. Nygaard V, Rødland EA, Hovig E. Methods that remove batch effects while retaining group differences may lead to exaggerated confidence in downstream analyses. *Biostatistics*. 2015;17(1):kxv027. doi:10.1093/biostatistics/kxv027

16. Müller C, Schillert A, Röthemeier C, et al. Removing Batch Effects from Longitudinal Gene Expression - Quantile Normalization Plus ComBat as Best Approach for Microarray Transcriptome Data. *PLoS One*. 2016;11(6):e0156594. doi:10.1371/journal.pone.0156594

17. Shaham U, Stanton KP, Zhao J, et al. Removal of Batch Effects using Distribution-Matching Residual Networks. 2016. doi:10.1093/bioinformatics/btx196

18. Chen C, Grennan K, Badner J, et al. Removing batch effects in analysis of expression microarray data: An evaluation of six batch adjustment methods. *PLoS One*. 2011;6(2). doi:10.1371/journal.pone.0017238

19. Johnson WE, Li C, Rabinovic A. Adjusting batch effects in microarray expression data using empirical Bayes methods. *Biostatistics*. 2007;8(1):118-127. doi:10.1093/biostatistics/kxj037

20. Leek JT, Johnson WE, Parker HS, Jaffe AE, Storey JD. The SVA package for removing batch effects and other unwanted variation in high-throughput experiments. *Bioinformatics*. 2012;28(6):882-883. doi:10.1093/bioinformatics/bts034

21. Bolstad BM, Irizarry RA, Astrand M, Speed TP. A comparison of normalization methods for high density oligonucleotide array data based on variance and bias. *Bioinformatics*. 2003;19(2):185-193. doi:10.1093/bioinformatics/19.2.185

22. Gagnon-Bartsch JA, Speed TP. Using control genes to correct for unwanted variation in microarray data. *Biostatistics*. 2012;13(3):539-552. doi:10.1093/biostatistics/kxr034

© 2021 Rychkov D et al. *JAMA* Network Open.

23. Oytam Y, Sobhanmanesh F, Duesing K, Bowden JC, Osmond-McLeod M, Ross J. Risk-conscious correction of batch effects: Maximising information extraction from high-throughput genomic datasets. *BMC Bioinformatics*. 2016;17(1):1-17. doi:10.1186/s12859-016-1212-5

24. Eisenberg E, Levanon EY. Human housekeeping genes, revisited. *Trends Genet*. 2013;29(10):569-574. doi:10.1016/j.tig.2013.05.010

25. Zhang Y, Li D, Sun B. Do housekeeping genes exist? *PLoS One*. 2015;10(5):1-22. doi:10.1371/journal.pone.0123691

26. Venet D, Dumont JE, Detours V. Most Random Gene Expression Signatures Are Significantly Associated with Breast Cancer Outcome. Rigoutsos I, ed. *PLoS Comput Biol*. 2011;7(10):e1002240. doi:10.1371/journal.pcbi.1002240

27. Freytag S, Gagnon-Bartsch J, Speed TP, Bahlo M. Systematic noise degrades gene co-expression signals but can be corrected. *BMC Bioinformatics*. 2015;16(1):309. doi:10.1186/s12859-015-0745-3

28. Jacob L. RUV for normalization of expression array data. 2015:1-16.

29. Risso D, Ngai J, Speed TP, Dudoit S. Normalization of RNA-seq data using factor analysis of control genes or samples. *Nat Biotechnol*. 2014;32(9):896-902. doi:10.1038/nbt.2931

30. Reese SE, Archer KJ, Therneau TM, et al. A new statistic for identifying batch effects in high-throughput genomic data that uses guided principal component analysis. *Bioinformatics*. 2013;29(22):2877-2883. doi:10.1093/bioinformatics/btt480

31. Tusher VG, Tibshirani R, Chu G. Significance analysis of microarrays applied to the ionizing radiation response. *Proc Natl Acad Sci*. 2001;98(9):5116-5121. doi:10.1073/pnas.091062498

32. Schwender H. Identifying differentially expressed genes with siggenes. (2003):1-30.

33. Goeman JJ, Solari A. Multiple hypothesis testing in genomics. *Stat Med*. 2014;33(11):1946-1978. doi:10.1002/sim.6082

34. Reiner-Benaim A. FDR control by the BH procedure for two-sided correlated tests with implications to gene expression data analysis. *Biometrical J*. 2007;49(1):107-126. doi:10.1002/bimj.200510313

35. Yu G, Wang L-G, Han Y, He Q-Y. clusterProfiler: an R Package for Comparing Biological Themes Among Gene Clusters. *Omi A J Integr Biol*. 2012;16(5):284-287. doi:10.1089/omi.2011.0118

36. Szklarczyk D, Gable AL, Lyon D, et al. STRING v11: Protein-protein association networks with increased coverage, supporting functional discovery in genome-wide experimental datasets. *Nucleic Acids Res*. 2019;47(D1):D607-D613. doi:10.1093/nar/gky1131

37. Aran D, Hu Z, Butte AJ. xCell: digitally portraying the tissue cellular heterogeneity landscape. *Genome Biol*. 2017;18(1):220. doi:10.1186/s13059-017-1349-1

38. Kuhn M. Building Predictive Models in R Using the caret Package. *J Stat Softw*. 2008;28(5):159-160. doi:10.18637/jss.v028.i05

39. Perez-Riverol Y, Kuhn M, Vizcaíno JA, Hitz M-P, Audain E. Accurate and fast feature selection workflow for high-dimensional omics data. Zou Q, ed. *PLoS One*. 2017;12(12):e0189875. doi:10.1371/journal.pone.0189875

© 2021 Rychkov D et al. *JAMA* Network Open.
